# Applying machine learning EEG signal classification to emotion‑related brain anticipatory activity

**DOI:** 10.12688/f1000research.22202.1

**Published:** 2020-03-10

**Authors:** Marco Bilucaglia, Gian Marco Duma, Giovanni Mento, Luca Semenzato, Patrizio E. Tressoldi

**Affiliations:** 1 Behavior and BrainLab, IULM, Milan, Italy; 2 Department of Developmental and Social Psychology (DPSS), Università degli Studi di Padova, Padova, Italy; 3 Department of General Psychology, Università degli Studi di Padova, Padova, Italy; 4 Science of Consciousness Research Group, Department of General Psychology, Università degli Studi di Padova, Padova, Italy

**Keywords:** EEG, brain anticipatory activity, machine learning, emotion

## Abstract

Machine learning approaches have been fruitfully applied to several neurophysiological signal classification problems. Considering the relevance of emotion in human cognition and behaviour, an important application of machine learning has been found in the field of emotion identification based on neurophysiological activity. Nonetheless, there is high variability in results in the literature depending on the neuronal activity measurement, the signal features and the classifier type. The present work aims to provide new methodological insight into machine learning applied to emotion identification based on electrophysiological brain activity. For this reason, we analysed previously recorded EEG activity measured while emotional stimuli, high and low arousal (auditory and visual) were provided to a group of healthy participants. Our target signal to classify was the pre-stimulus onset brain activity. Classification performance of three different classifiers (linear discriminant analysis, support vector machine and k-nearest neighbour) was compared using both spectral and temporal features. Furthermore, we also contrasted the classifiers’ performance with static and dynamic (time evolving) features. The results show a clear increase in classification accuracy with temporal dynamic features. In particular, the support vector machine classifiers with temporal features showed the best accuracy (63.8 %) in classifying high vs low arousal auditory stimuli.

## Introduction

In last decades, the vision of the brain has moved from a passive stimuli elaborator to an active reality builder. In other words, the brain is able to extract information from the environment, building up inner models of external reality. These models are used to optimize the behavioural outcome when reacting to upcoming stimuli ^1– 4^.

One of the main theoretical models assumes that the brain, in order to regulate body reaction, runs an internal model of the body in the world, as described by embodied simulation framework ^5^. A much investigated hypothesis is that the brain functions as a Bayesian filter for incoming sensory input; that is, it activates a sort of prediction based on previous experiences about what to expect from the interaction with the social and natural environment, including emotion ^6^. In light of this, it is possible to consider emotions, not only as a reaction to the external world, but also as partially shaped by our internal representation of the environment, which help us to anticipate possible scenarios and therefore to regulate our behaviour.

The construction model of emotion ^7^ argues that the human being actively builds-up his/her emotions in relation to the everyday life and social context in which they are placed. We actively generate a familiar range of emotions in our reality, based on their usefulness and relevance in our environment. In this scenario, in a familiar context we are able to anticipate which emotions will be probably elicited, depending on our model. As a consequence, the study of the anticipation of/preparation for forthcoming stimuli may represent a precious window for understanding the individual internal model and emotion construction process, resulting in a better understanding of human behaviour.

A strategy to study preparatory activity could be related to the experimental paradigm in which cues are provided regarding the forthcoming stimuli, allowing the investigation of the brain activity dedicated to the elaboration of incoming stimuli ^8, 9^. A cue experiment to predict the emotional valence of the forthcoming stimuli showed that the brain’s anticipatory activation facilitates, for example, successful reappraisal via reduced anticipatory prefrontal cognitive elaboration and better integration of affective information in the paralimbic and subcortical systems ^10^. Furthermore, preparation for forthcoming emotional stimuli also has relevant implications for clinical psychological conditions, such as mood disorders or anxiety ^11, 12^.

Recently, the study of brain anticipatory activity has been extended to statistically unpredictable stimuli ^13– 15^, providing experimental hints of specific anticipatory activity before stimuli are randomly presented. Starting from the abovementioned studies, we focused on the extension of brain anticipatory activity to statistically unpredictable emotional stimuli.

According to the so called dimensional model, emotion can be defined in terms of three different attributes (or dimensions): valence, arousal and dominance. Valence measures the positiveness (ranging from unpleasant to pleasant), arousal measures the activation level (ranging from boredom to frantic excitement) and dominance measures the controllability (i.e. the sense of control) ^16^.

Emotions can be estimated from various physiological signals ^17^, such as via skin conductance, electrocardiogram (ECG) and electroencephalogram (EEG). The latter has received a considerable amount of attention in the last decade, introducing several machine learning and signal processing techniques, originally developed in other contexts, such as brain computer interfaces ^18^. Emotion recognition has been re-drawn as a machine learning problem, where proper EEG related features are used as inputs to specific classifiers.

The most common features belong the spectral domain, in the form of spectral powers in delta, theta, alpha and gamma bands ^19^, as well as power spectral density (PSD) bins ^20^. The remaining belong to the time domain, in the form of event-related de/synchronizations (ERD/ERS) and event-related potentials (ERP) ^19^, as well as shape related indices such as the Hjorth parameters and the fractal dimension ^20^.

The most commonly used classifier is the support vector machine (SVM) with the radial basis function (RBF) kernel, followed by the k-nearest neighbour (kNN) and the linear discriminant analysis (LDA) ^19, 20^. Finally, most of the classifiers are implemented as non-adaptive (i.e. static) ^19^, in contrast to the dynamic versions that take into account the temporal variability of the features ^21^.

The classification performances are very variable because of the different features and classifiers adopted. The following examples are taken from 19 - in particular, from the subset (17 out of 63) of reviewed papers that focused on arousal classification. Using an SVM (RBF kernel) and spectral features (e.g. short-time Fourier transform), Lin and colleagues obtained 94.4% accuracy (i.e. percentage of corrected classification) ^22^, while using similar spectral features (e.g. PSD) and classifier (SVM with no kernel), Koelstra and colleagues obtained an accuracy of 55.7% ^23^. Liu and Sourina obtained an accuracy of 76.5% using temporal features (e.g. fractal dimension) with an SVM (no kernel) ^24^, while Murugappan and Murugappan obtained a an accuracy of 63% using similar temporal features and an SVM with a polynomial kernel ^25^. Finally, Thammasan and collegues obtained an accuracy of 85.3% using spectral features (e.g. PSD), but with a kNN (with k=3) ^26^. All the classifiers were static.

The purpose of the present work is to provide new methodological advancements on the machine learning classification of emotions, based on the brain anticipatory activity. For this purpose, we compared the performances of tree different classifiers (namely LDA, SVM, kNN) trained using two types of EEG features (namely, spectral and temporal). In addition, each classifier was dynamically trained, to take into account the temporal variability of the features. The results provide useful insights regarding the best classifier-features configuration to better discriminate emotion-related brain anticipatory activity.

A more detailed description of the machine learning algorithms is provided as *Extended data* ^27^ *.*

### Classification performances

In introducing pattern recognition, we underlined that the classifiers are built using a set of previously annotated class-prototypical features for the training set. It is common practice to extract from the training set a subset of annotated features (the test set) and use it to evaluate the performances of the trained classifiers – but not to train it.

Since the training set is limited, the specific train/test splitting introduce a bias in both the training and performance evaluation. This can be avoided following the so-called *k* -fold cross validation scheme. The original training set *D* is partitioned into *k* disjoint and equal sized sets, $D=\cup_{i=1}^{k}D_{k}$ . The classifier is then trained *k*-times using, each time, as the test set a different partition *D _j_* and as the training set the remaining $\cup_{i\neq j}D_{i}.$ Finally, the overall performance is computed as the average over the *k* single performances ^28^ (pp. 483–485).

With the general term performance, we mostly refer to the classification accuracy *ACC*, defined as the ratio between the number of correctly classified features and the total number of features. Introducing the chance-level accuracy *ACC*_0_ as the ratio between the number of features for each class (i.e. how balanced is the training set), we can additionally define as performance the Kappa statistic: *κ* = ( *ACC* – *ACC*_0_)/ *ACC*_0_^29^.

Compared to *ACC*, *κ* is a more robust performance measure, since it is normalized by the class unbalances. Another solution to take into account the class unbalances, is to compare (using for example a t-test) the *k* cross-validated accuracies against *k* random accuracies, obtained from a random classifier ^29– 35^.

### Dynamic classifiers

To classify a time-varying signal (i.e. to perform a dynamic classification), an ordered sequence of features ${\text{\{}x_{i}\text{\}}}_{i=1}^{N}$ (i.e. temporal features), corresponding to *N* adjacent temporal windows, is extracted. The temporal features are fed into either “dynamic” classifiers, such as the Hidden Markow Model (HMM) ^21^, or an ordered sequence of “static” classifiers ${\text{\{}f_{i}\text{\}}}_{i=1}^{N}$ ^36– 39^. The former fully takes into account the signal’s temporal variability, since it uses the entire sequence during the training phase. The latter train each static classifier *f _i_*, using only the corresponding features **x**_*i*_, but provides an ordered sequence of accuracies ${\left\{ACC_{i}\right\}}_{i=1}^{N}$ . where each *ACC _i_* corresponds to *f _i_*.

### Feature selection

As stated in the previous sections, the curse of dimensionality arises when the number of available training features is small compared to the feature dimension *m*. In such situations, the parameter estimation becomes problematic (see for example the problem of the singularity of the estimated covariance matrix described in the *LDA* sub-section) and the trained classifier usually underperforms.

As a rule of thumb, the number of training features *N* should be an exponential function of the dimensionality (e.g. *N* = 10 ^*m*^), with the ratio growing with the complexity of the classifiers ^40^. By fixing the feature dimension *m*, linear classifiers require, for example, a less numerous training set. Additionally, even with an adequate training set, feature dimensionality impacts on both the training and classification speed. In fact, as stated in the *SVM* sub-section, linear classification requires *O*( *m*) multiplications and sums to compute each scalar product. Reducing the feature dimensionality by means of so called feature selection algorithms, a classifier can be made more robust (i.e. less sensitive to the curse of dimensionality) and efficient (in terms of computational speed).

Feature selection can be broadly described as a mapping function $s:\;{\mathbb{R}}^{m}\to {\mathbb{R}}^{n}$ such as:

$$s(\text{x})\;=\;{\left(x_{s_{1}},x_{s_{2}},\;...\;,\;x_{s_{n}}\right)}^{T}\;(13)$$

where *n* < *m* and { *s*_1_, *s*_2_, ... , *s*_n_} ⊂ {1, 2, ... , *m*}. In other words, a feature selection algorithm performs a projection of the original feature vector onto a lower dimensional subspace defined by a subset of scalar features. The best subspace, as selected among all the possible 2 ^*m*^, should not significantly decrease the classification performances, both globally (i.e. how features are classified overall) and locally (i.e. how the single feature is classified) ^41^

Feature selection algorithms can be broadly grouped according to the following criteria ^42^:

1. Label information. Supervised algorithms take into account the class information, while unsupervised algorithms do not, in assigning the training features as belonging to the same class.

2. Search strategy. Filter algorithms (also known as classifier-independent) are based on a two-step “ranking and selecting” criterium: scalar features are first ranked according to a proper criterion; then only the “best” ones are selected. Wrapper methods (also known as classifier dependent methods) use the selected classifier, following an “ad hoc” approach: the selected scalar features are those that give the best classification performance

An example of a supervised filter algorithm is the biserial correlation coefficient. Given a training set *D* composed by *N*_+_ features belonging to the class +1 and *N*_–_ features belonging to the class –1, the biserial correlation coefficient for the *k*-th scalar feature *x _k_* is given by 43:

$$r_{k}^{2}=\frac{\sqrt{N_{+}N_{-}}}{N_{+}\;+\;N_{-}}\frac{m\left(x_{k}^{+}\right)-m\left(x_{k}^{-}\right)}{s\left(x_{k}\right)}\;(13)$$

where *m*(·), *s*(·) are the sample mean and sample standard deviation operators, respectively, and $x_{k}^{+}$ , $x_{k}^{-}$ are the subset of *x _k_* belonging to the classes +1 and –1, respectively. The total feature score is obtained by summing the *m* coefficients of each scalar feature *x _k_*. Once the scores $r_{k}^{2}$ are sorted in descending order, the feature selection is made simply by selecting the first scalar features whose summing score get a percentage (e.g. 95%) of the total feature score.

## Methods

### Ethical statement

The data of the present study were obtained in the experiment described in 37, which was approved by the Ethical Committee of the Department of General Psychology, University of Padova (No. 2278). Before taking part in the experiment, each subject gave his/her informed consent in writing after having read a description of the experiment. In line with department policies, this re-analysis of an original study approved by the ethics committee did not require new ethical approval.

### Stimuli and experimental paradigm

In the present study, we reanalysed the EEG data ^27^ of the experiment described in 37, applying an original static and dynamic features selection and classification by using the three different algorithms explained above.

A more detailed description of the experimental design is available in the original study. Here we describe only the main characteristics.

Two sensory categories of stimuli (i.e. visual and auditory), were extracted according to their arousal value from two standardized international archives. Visual stimuli consisted of pictures of 28 faces, 14 neutral faces and 14 fearful faces were extracted from the NIMSTIM archive ^44^, whereas auditory stimuli consisted of 28 sounds, and 14 low- and 14 high-arousal sounds were chosen from the International Affective Digitized Sounds (IADS) archive ^45^.

**Figure 1.**
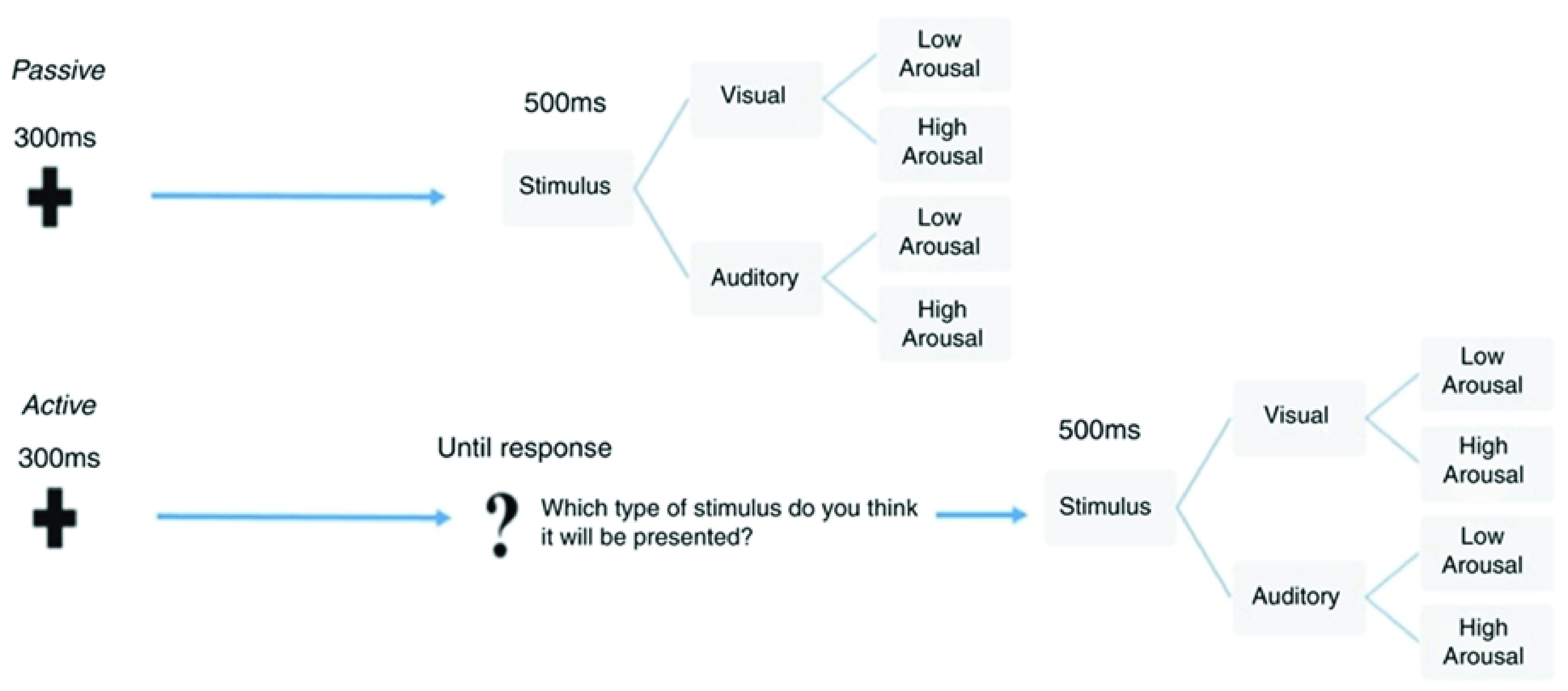
Experimental tasks.

To all 28 adult healthy participants, two different experimental tasks, which were delivered in separate blocks were presented. The two tasks are described in Figure 1, which illustrates the sequence of events and the temporal trial structure relative to the passive (top) and the active (bottom) tasks. Within each task, the stimuli were randomly presented and equally distributed according to either sensory category (faces or sounds) and arousal level (high or low). Full details of these tasks have been described previously in 37.

### EEG recording

During the entire experiment, the EEG signal was continuously recorded using a Geodesic high density EEG system (EGI GES-300) through a pre-cabled 128-channel HydroCel Geodesic Sensor Net (HCGSN-128) referenced to the vertex (CZ), with a sampling rate of 500 Hz. The impedance was kept below 60kΩ for each sensor. To reduce the presence of EOG artefacts, subjects were instructed to limit both eye blinks and eye movements, as much as possible.

### EEG preprocessing

The continuous EEG signal was off-line band-pass filtered (0.1–45Hz) using a Hamming windowed sinc finite impulse response (FIR) filter (order = 16500) and then downsampled at 250 Hz. The EEG was epoched starting from 200 ms before the cue onset and ending at the stimulus onset. The initial epochs were 1300 ms long from the cue onset, including 300 ms of cue/fixation cross presentation and 1000 ms of interstimulus interval (ISI).

All epochs were visually inspected to remove bad channels and rare artefacts. Artefact-reduced data were then subjected to independent component analysis (ICA) ^45^. All independent components were visually inspected, and those that related to eye blinks, eye movements, and muscle artefacts, according to their morphology and scalp distribution, were discarded. The remaining components were back-projected to the original electrode space to obtain cleaner EEG epochs.

The remaining ICA-cleaned epochs that still contained excessive noise or drift (±100 μV at any electrode) were rejected and the removed bad channels were reconstructed. Data were then re-referenced to the common average reference (CAR) and the epochs were baseline-corrected by subtracting the mean signal amplitude in the pre-stimulus interval. From the original 1300 ms long epochs, final epochs were obtained only from the 1000 ms long ISI.

### Static spectral features

From each epoch and each channel *k*, the PSD was estimated by a Welch’s periodogram using 250 points long Hamming’s windows with 50% overlapping. PSD was first log transformed to compensate the skewness of power values ^46^, then the spectral bins corresponding to alpha, beta and theta bands – defined as 13~30 *Hz*, 6~13 *Hz* and 4~6 *Hz*, respectively ^47^ – were summed together. Finally, alpha, beta and theta total powers were computed as:

$$\beta_{tot}^{k}=\sum_{i\in [13;30]}PSD^{k}(i)\;$$

$$\alpha_{tot}^{k}=\sum_{i\in [6;13]}PSD^{k}(i)\;(14)$$

$$\theta_{tot}^{k}=\sum_{i\in [4;6]}PSD^{k}(i)\;$$

As a measure of emotional arousal, we computed the ratio between beta and alpha total powers $\beta_{tot}^{k}/\alpha_{tot}^{k}\;\;\;$ ^48^, while to measure cognitive arousal, we computed the ratio between beta and theta total powers $\theta_{tot}^{k}/\beta_{tot}^{k}\;\;\;$ ^49^.

For each epoch, the feature (with a dimensionality of 256) was obtained, concatenating the beta-over-alpha and beta-over-theta ratio of all the channels:

$$v≐\left[\frac{\beta_{tot}^{1}}{\alpha_{tot}^{1}},\frac{\theta_{tot}^{1}}{\beta_{tot}^{1}},\frac{\beta_{tot}^{2}}{\alpha_{tot}^{2}},\frac{\theta_{tot}^{2}}{\beta_{tot}^{2}},...,\frac{\beta_{tot}^{128}}{\alpha_{tot}^{128}},\frac{\theta_{tot}^{128}}{\beta_{tot}^{128}}\right]\;(15)$$

### Static temporal features

It has been previously shown that arousal level (high or low) can be estimated from the contingent negative variation potentials ^37^. The feature extraction procedure, therefore, follows the classical approach for event-related potentials ^50^. Each epoch from each channel was first band pass filtered (0.05~10 *Hz*) using a zero-phase 2 ^nd^ order Butterworth filter and decimated to a sample frequency of 20 *Hz*. EEG signal was thus normalized (i.e. z-scored) according to the temporal mean and the temporal standard deviation:

$$x_{i}(t_{k})=({\tilde{x}}_{i}(t_{k})-m_{i})/s_{i}$$

where ${\tilde{x}}_{i}(t_{k})$ is the raw signal from i-th channel at time point *t _k_*, and *m _i_* and *s _i_* are, respectively, the temporal mean and the temporal standard deviation of the i-th channel. For each epoch, the feature (with a dimensionality of 2560) was obtained, concatenating all normalized signal from each channel:

$$v≐\left[x_{1}(t_{1}),x_{1}(t_{2}),...,x_{1}(t_{20}),...,x_{128}(t_{1}),x_{128}(t_{2}),...,x_{128}(t_{20})\right]\;(16)$$

### Dynamic features

Each epoch was partitioned into 125 temporal segments, 500 ms long and shifted by 1/250 s (one sample). Within each time segment, we extracted the dynamic spectral and temporal features, following the same approaches described in *Static spectral features* and *Static temporal features* sub-sections, respectively. Dynamic temporal features had a dimensionality of 1280, corresponding to 0.5 × 20 = 10 samples per channel. Dynamic spectral features had the same dimensionality as their static counterparts (256), but the Welch’s periodogram was computed using a 16 points long Hamming’s window (zero-padded to 250 points) with 50% overlapping.

### Feature reduction and classification

The extracted features (both static and dynamic) were grouped according to the stimulus type (sound or image) and the task (active or passive), in order to classify the group-related arousal level (high or low). A total of four binary classification problems (high arousal vs low arousal) were performed: active image (Ac_Im), active sound (Ac_So), passive image (Ps_Im) and passive sound (Ps_So).

Static features were reduced by means of the biserial correlation coefficient *r* ^2^ with the threshold set at 90% of the total feature score. In order to identify the discriminative power of each EEG channel, a series of scalp plots (one for each feature type and each group) of the coefficients were drawn. Since each channel is associated with *N* > 1 features (as well as *N* *r* ^2^ coefficients), the coefficients (one coefficient for each channel) are calculated as a mean value. In other words, spectral and temporal features had two and 20 scalar features, respectively, for each EEG channel. To compute their scalp plots, we averaged 2 and 20 *r* ^2^ coefficients of each channel. To enhance the visualization of the plots, the coefficients were finally normalized to the total score and expressed as a percentage.

Each classification problem was addressed by the mean of three classifiers: LDA with pseudo-inverse covariance matrix; soft-margin SVM with penalty parameter *C* = 1 and RBF kernel; and kNN with Euclidean distance and k=1. Additionally, a random classifier, giving a uniform pseudo-random class (Pr{HA} = Pr{LA} = 0.5), served as a benchmark ^29^. The accuracy of the classifiers was measured, repeating 10 times for a 10-fold cross-validation scheme. The feature selection was computed within each cross-validation step, to avoid overfitting and reduce biased results ^43^.

For each group (Ac_Im, Ac_So, Ps_Im, Ps_So) and each feature type (static spectral, static temporal), the classification produced a 10 × 4 matrix containing the mean accuracies (one for each of the 10-fold cross-validation repetitions) of each classifier.

Dynamic features were reduced and classified similarly to the static ones. For each temporal segment, the associated features were reduced by means of the biserial correlation coefficient (threshold at 90%) and the classifiers (SVM, kNN, LDA and random) were evaluated using a 10-fold cross-validation scheme – repeated 10 times.

For each group, each feature type (dynamic spectral, dynamic temporal), each temporal segment and each classifier, the classification produced 10 sequences of mean accuracies ${\left\{ACC_{i}\right\}}_{i=1}^{125}$ – one for each repetition of the 10-fold cross-validation scheme.

### Data analysis

The syntax in MATLAB used for all analyses is available on GitHub along with the instructions on how to use it (see *Software availability*) ^51^. The software can also be used with the open source program Octave.

### Statistical analysis

The results of the static classifications were compared against the benchmark classifier by means of a two-sample t-test (right tail).

The results of dynamic classifications (i.e. based on dynamic spectral or dynamic temporal features) were compared following a segment-by-segment approach. For each group, the accuracy sequences of the dynamic classifiers (SVM, kNN and LDA) were compared with the benchmark accuracy sequence. Each sample $ACC_{i}^{k}$ , with *k* = {SVM, kNN, LDA}, was tested against $ACC_{i}^{\text{Random}}$ by means of two-sample t-tests (right tail). The corresponding p-value sequences ${\left\{p_{i}^{k}\right\}}_{i=1}^{125}$ were Bonferroni-Holm corrected for multiple comparisons. Finally, the best accuracy point was detected as the left extreme of the temporal window corresponding to the highest significant accuracy.

## Results

### Static features

**Figure 2.**
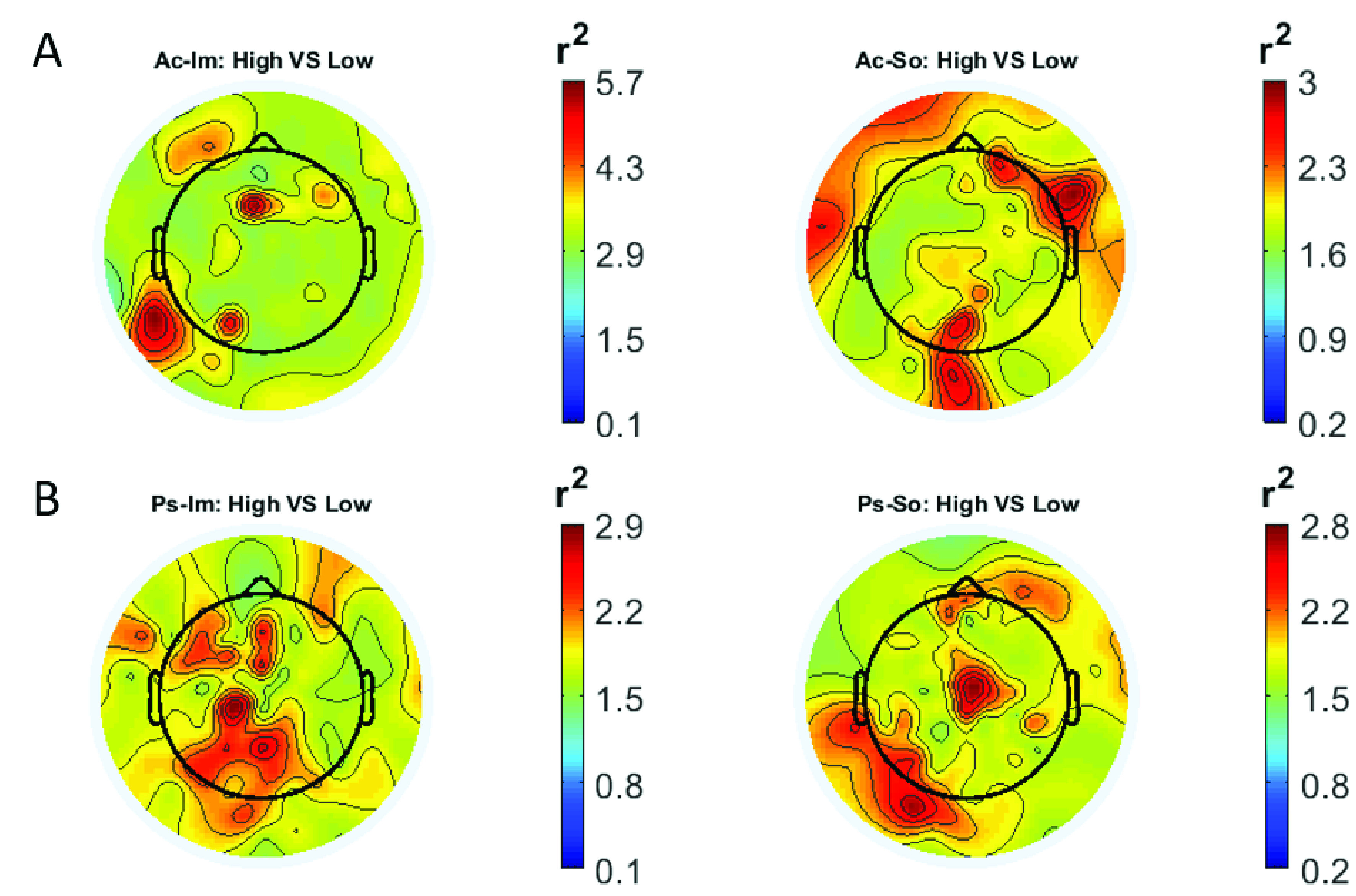
Spectral features. Scalp distribution of the *r* ^2^ coefficients (normalized to the total score and expressed as percentage) grouped for tasks and stimulus type. ( **a**) Active task: left Image, right Sound; ( **b**) Passive task: left Image, right Sound.

**Figure 3.**
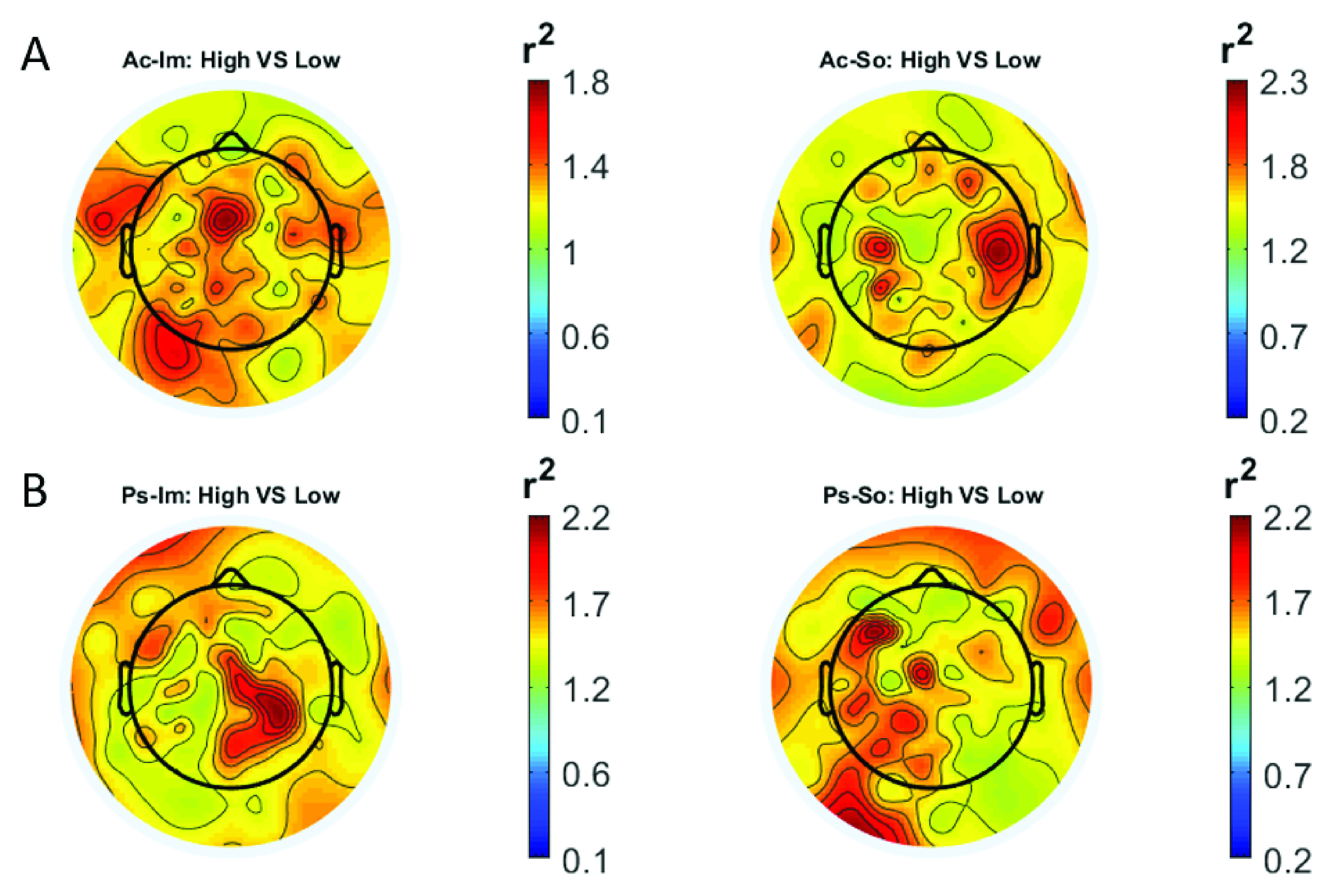
Temporal features. Scalp distribution of the *r* ^2^ coefficients (normalized to the total score and expressed as percentage), grouped for tasks and stimulus type. ( **a**) Active task: left Image, right Sound; ( **b**) Passive task: left Image, right Sound.

In Figure 2 and Figure 3, the scalp distributions of *r* ^2^ coefficients for each binary static classification problem, grouped for feature (spectral, temporal) and groups (Ps_Im, Ps_So, Ac_Im, Ac_So), are shown.

The temporal feature gave the most consistent topographical pattern, showing that the regions that best differentiate between high vs low stimuli (auditory and visual) were located over the central-parietal electrodes, whereas a more diffuse pattern in the scalp topography emerged for the spectral features.

**Figure 4.**
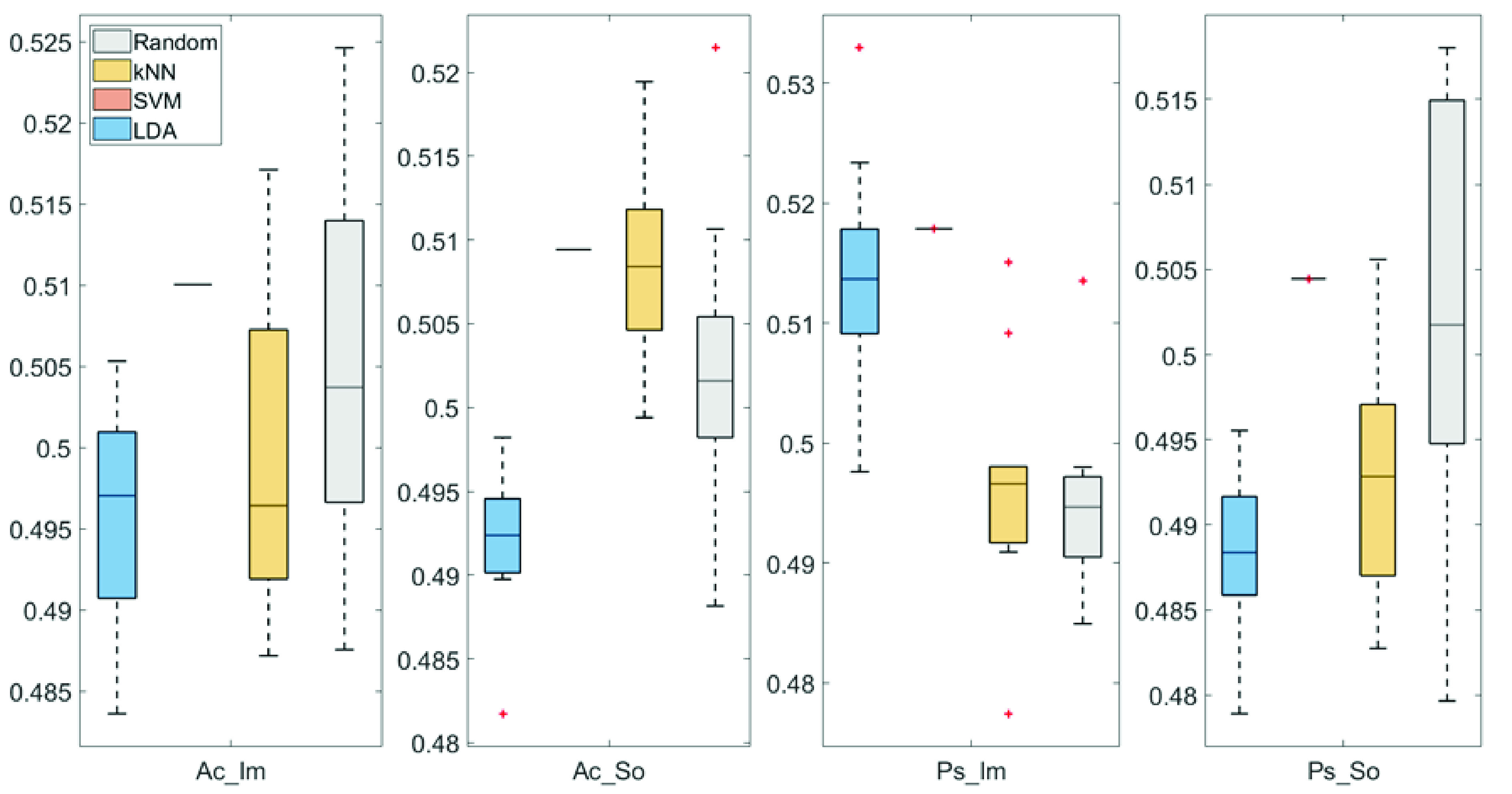
Box-plots of the accuracies of the static spectral classifications. From left: Active Image (Ac_Im), Active Sound (Ac_So), Passive Image (Ps_Im) and Passive Sound (Ps_So).

**Figure 5.**
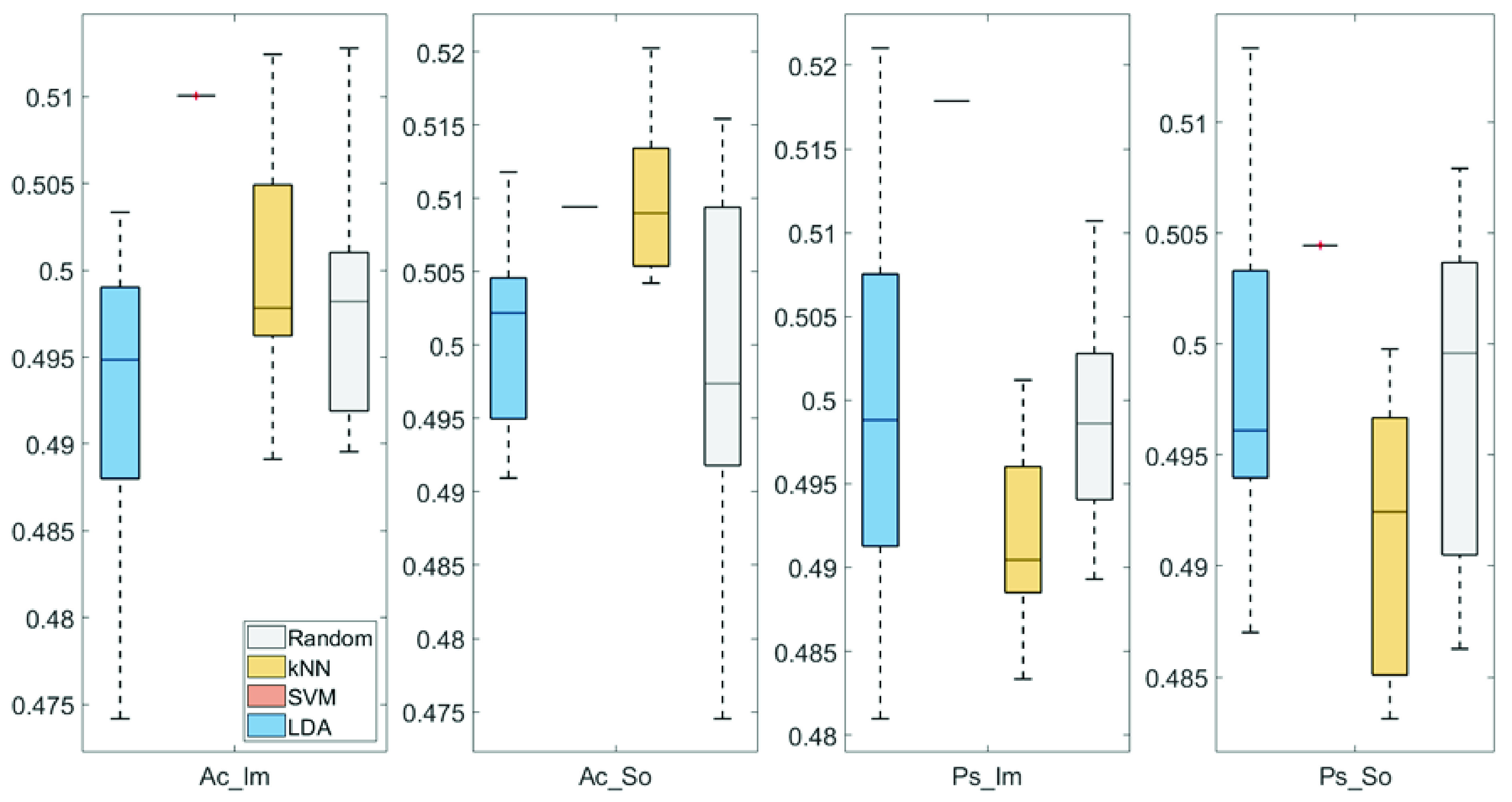
Box-plots of the accuracies of the static temporal classifications. From left: Active Image (Ac_Im), Active Sound (Ac_So), Passive Image (Ps_Im) and Passive Sound (Ps_So).

In Figure 4 and Figure 5, box plots of the accuracies of static temporal and spectral classifications, grouped for condition, are shown. Note that SVM accuracies (the 2 ^nd^ boxplot from the left) are always shown as lines because the accuracies were constant within each cross-validation step (see also Table 1, Table 2 and Table 3).

**Table 1.** Static features. Ordered accuracies grouped for classifier, feature and group.

Classifier	Accuracy	Feature	Group
SVM	51.80%	Spectral	Ps_Im
LDA	51.40%	Spectral	Ps_Im
kNN	51%	Temporal	Ac_So
kNN	50.90%	Spectral	Ac_So
SVM	50.90%	Spectral	Ac_So
SVM	50.90%	Temporal	Ac_So
SVM	50.40%	Temporal	Ps_So

SVM, support vector machine; LDA, linear discriminant analysis; kNN, k-nearest neighbour.

**Table 2.** Mean (M) and standard deviations (SD) of the accuracies of the static spectral classifications. Active Image (Ac_Im), Active Sound (Ac_So), Passive Image (Ps_Im) and Passive Sound (Ps_So).

Group	LDA	SVM	kNN	Random
Ac_Im	M=0.496, SD=0.007	M=0.510, SD=0.000	M=0.500, SD=0.010	M=0.505, SD=0.011
Ac_So	M=0.492, SD=0.004	M=0.509, SD=0.000	M=0.509, SD=0.007	M=0.503, SD=0.009
Ps_Im	M=0.514, SD=0.010	M=0.518, SD=0.000	M=0.496, SD=0.010	M=0.495, SD=0.008
Ps_So	M=0.488, SD=0.005	M=0.504, SD=0.000	M=0.493, SD=0.007	M=0.503, SD=0.013

SVM, support vector machine; LDA, linear discriminant analysis; kNN, k-nearest neighbour.

**Table 3.** Mean (M) and standard deviations (SD) of the accuracies of the static temporal classifications. Active Image (Ac_Im), Active Sound (Ac_So), Passive Image (Ps_Im) and Passive Sound (Ps_So).

Group	LDA	SVM	kNN	Random
Ac_Im	M=0.492, SD=0.010	M=0.510, SD=0.000	M=0.500, SD=0.008	M=0.498, SD=0.007
Ac_So	M=0.501, SD=0.007	M=0.509, SD=0.000	M=0.510, SD=0.006	M=0.498, SD=0.012
Ps_Im	M=0.500, SD=0.012	M=0.518, SD=0.000	M=0.492, SD=0.005	M=0.499, SD=0.006
Ps_So	M=0.499, SD=0.008	M=0.504, SD=0.000	M=0.492, SD=0.006	M=0.498, SD=0.008

SVM, support vector machine; LDA, linear discriminant analysis; kNN, k-nearest neighbour.

Note that all the accuracies refer to the same static classification problem (high arousal vs low arousal), performed using different classifiers (SVM, LDA, kNN) and features (spectral, temporal), on different groups (Ps_Im, Ps_So, Ac_Im, Ac_So).

Using spectral features, in only two groups did some classifiers show an accuracy greater than the benchmark. In the Ac_So group, *ACC _SVM_* = 50.9% (t(18)=2.371, p=0.015) and *ACC _kNN_* = 50.9% (t(18)=1.828, p=0.042), while for Ps_Im, *ACC _LDA_* = 51.4% (t(18)=4.667, p<0.001) and *ACC _SVM_* = 51.8% (t(18)=9.513, p<0.001).

Using temporal features, in all the groups some classifiers showed an accuracy greater than the benchmark. In the Ac_So group, *ACC _SVM_* = 50.9% (t(18)=2.907, p=0.005) and *ACC _kNN_* = 51% (t(18)=2.793, p=0.006) and in the Ps_So group, *AAC _SVM_* = 50.4% (t(18)=9.493, p<0.001).

### Dynamic features

**Figure 6.**
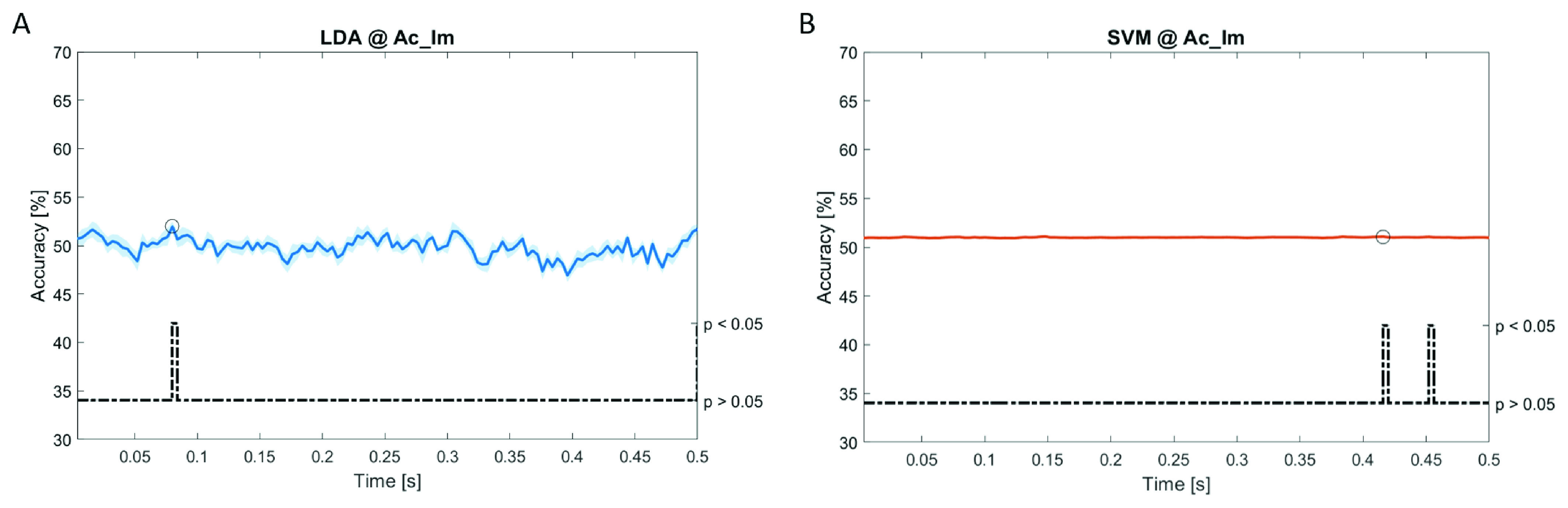
Spectral dynamic features. Accuracy (mean value, coloured line; standard deviation, shaded line) and p-values (black dotted line) in Ac_Im group for LDA ( **a**) and SVM ( **b**) classifiers.

In Figure 6– Figure 12, the results of the significant dynamic classifications are shown. In the upper section of the plots, the mean (bold line) and the standard deviation (shaded) of the accuracy sequence are shown. In the lower section of the plot (black dashed line), the Bonferroni-Holm corrected p-values sequence, discretized (as a stair graph) as significant (p<0.05) or non-significant (p>0.05) is shown.

**Figure 7.**
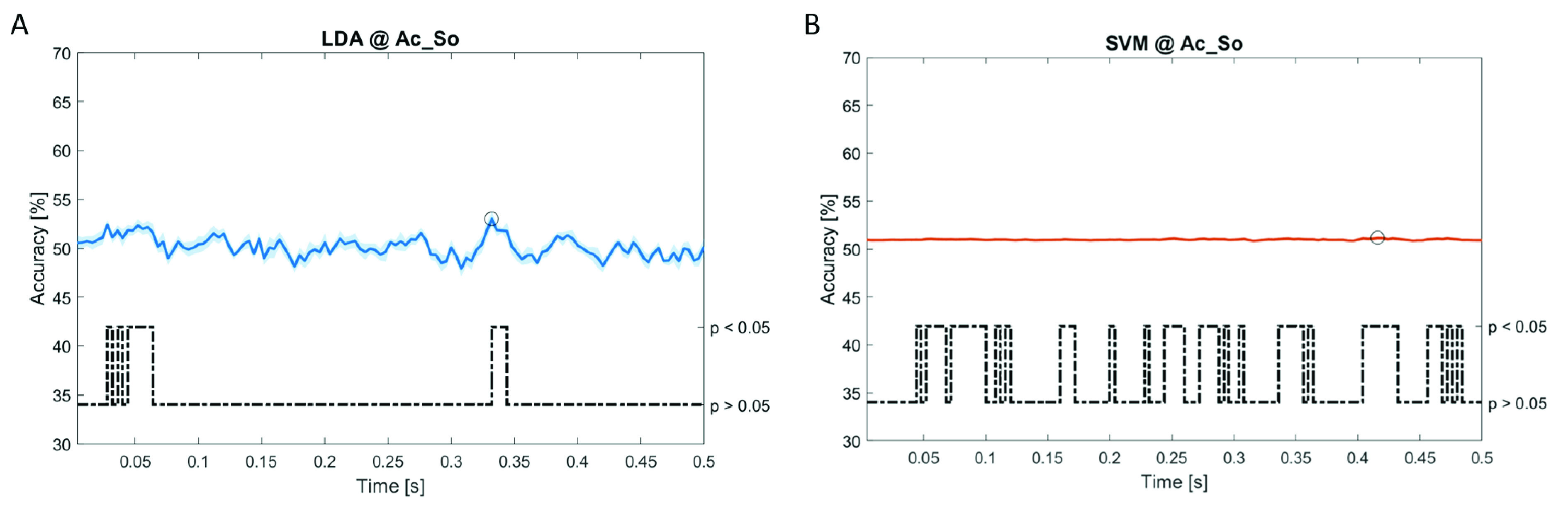
Spectral dynamic features. Accuracy (mean value, coloured line; standard deviation, shaded line) and p-values (black dotted line) in Ac_So group for LDA ( **a**) and SVM ( **b**) classifiers.

**Figure 8.**
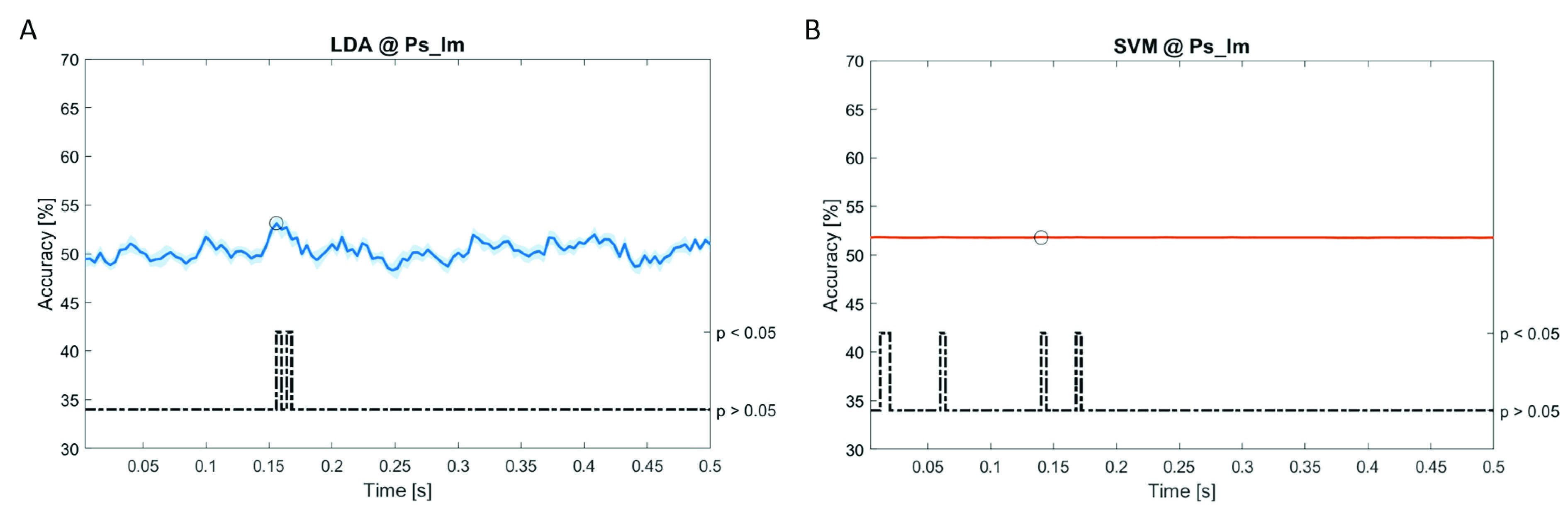
Spectral dynamic features. Accuracy (mean value, coloured line; standard deviation, shaded line) and p-values (black dotted line) in Ps_Im group for LDA ( **a**) and SVM ( **b**) classifiers.

**Figure 9.**
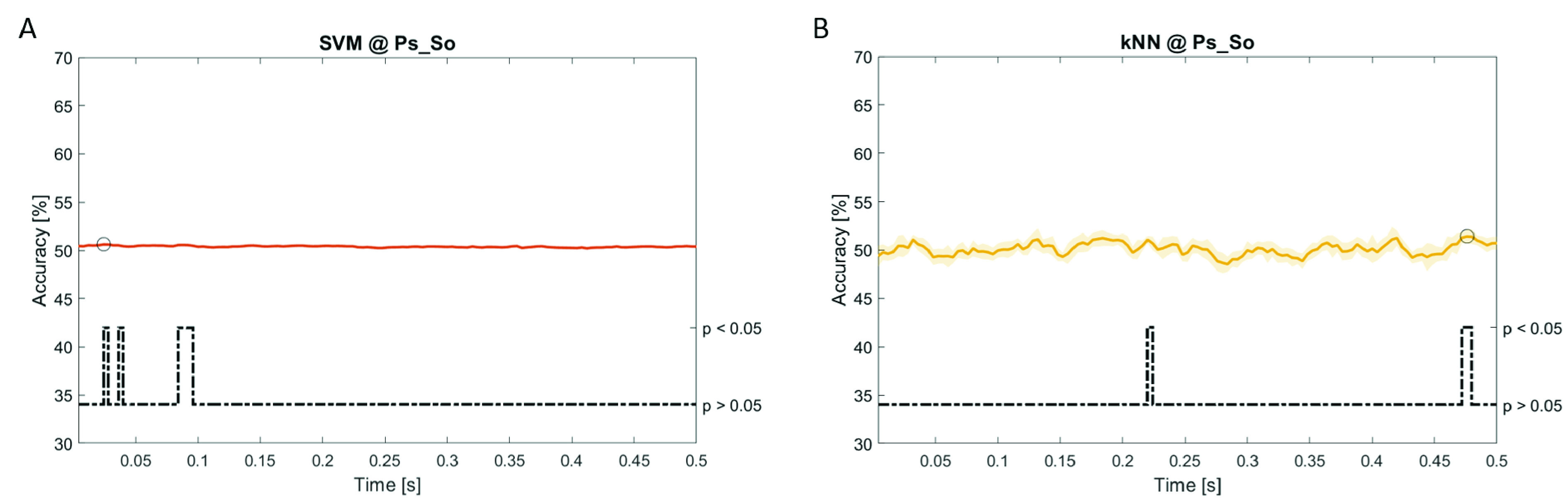
Spectral dynamic features. Accuracy (mean value, coloured line; standard deviation, shaded line) and p-values (black dotted line) in Ac_So group for SVM ( **a**) and kNN ( **b**) classifiers.

**Figure 10.**
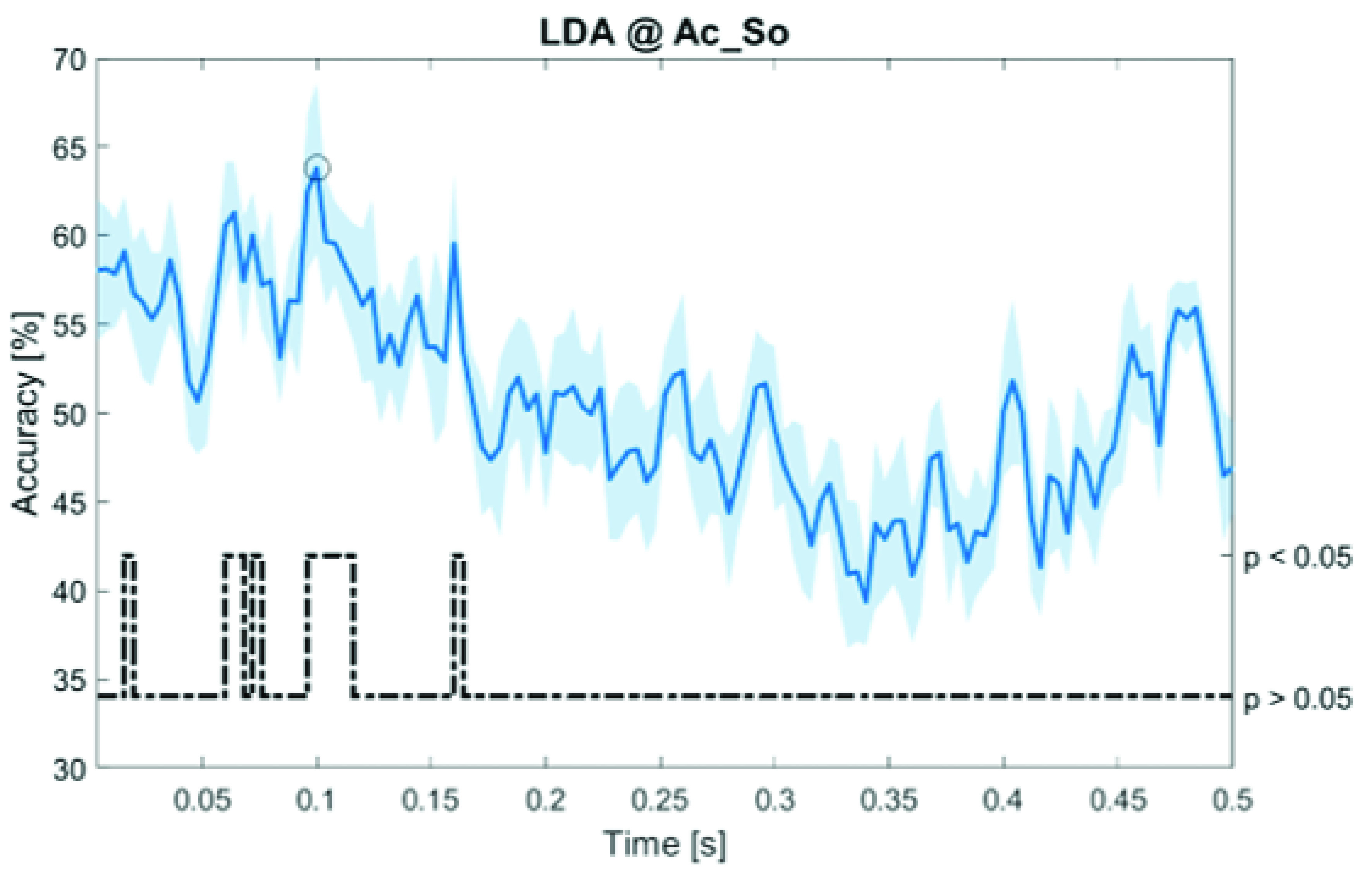
Temporal dynamic features. Accuracy (mean value, coloured line; standard deviation, shaded line) and p-values (black dotted line) in Ac_So group for LDA classifier.

**Figure 11.**
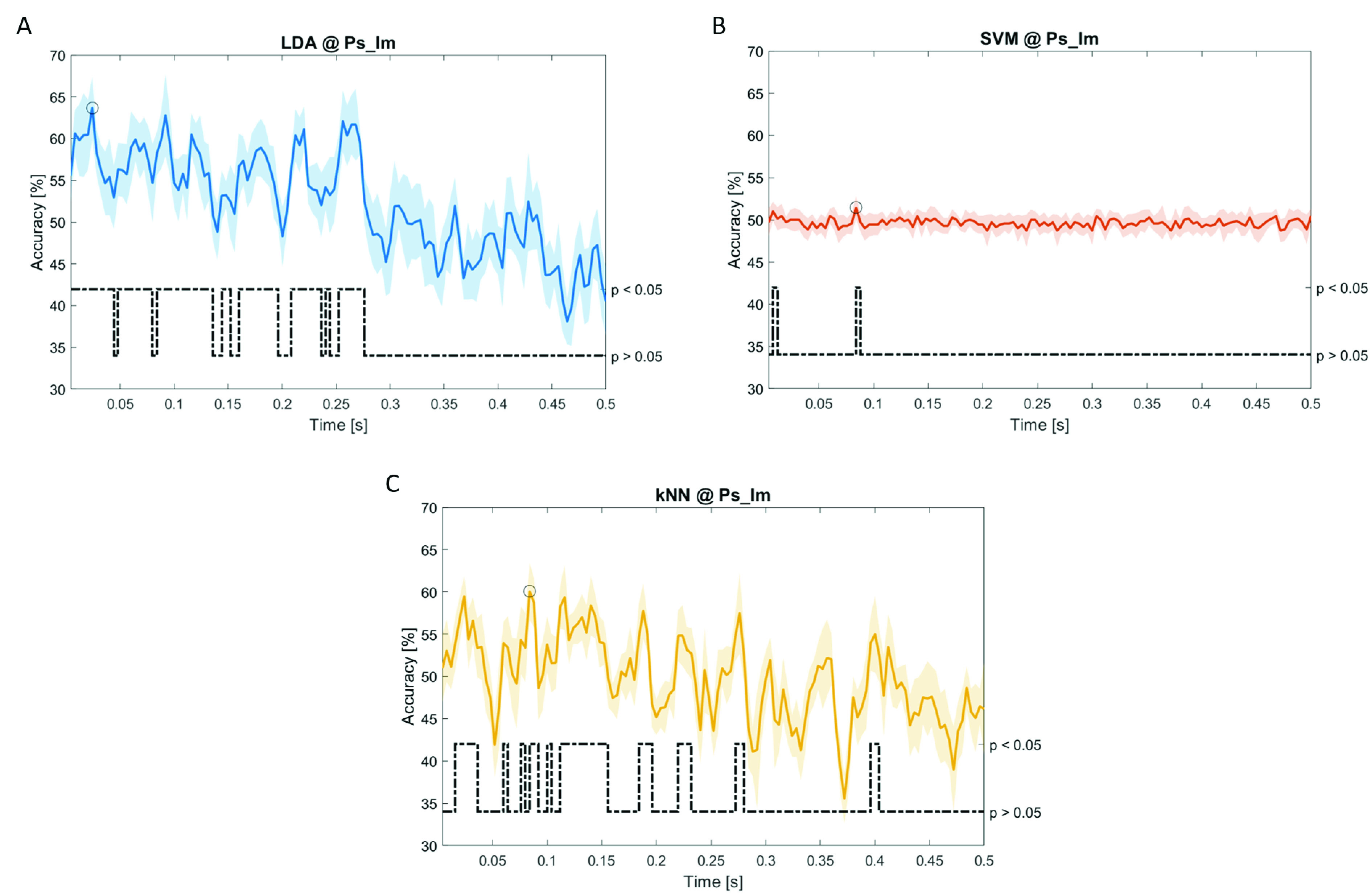
Temporal dynamic features. Accuracy (mean value, coloured line; standard deviation, shaded line) and p-values (black dotted line) in Ps_Im group for LDA ( **a**), SVM ( **b**) and kNN ( **c**) classifiers.

**Figure 12.**
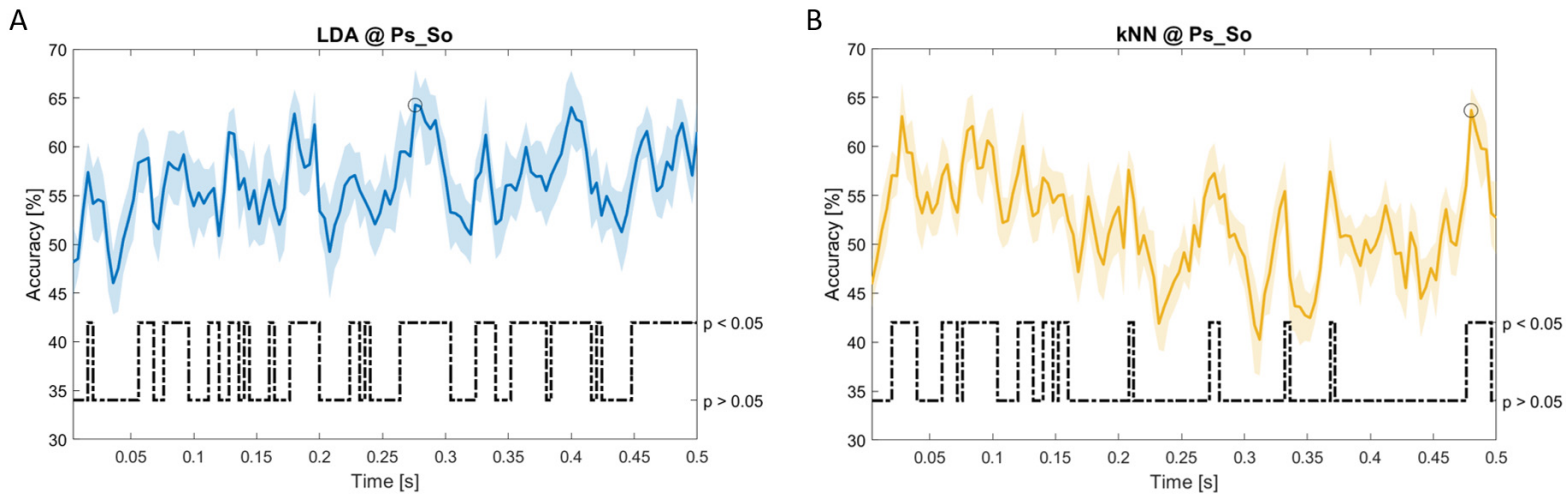
Temporal dynamic features. Accuracy (mean value, coloured line; standard deviation, shaded line) and p-values (black dotted line) in Ps_So group for LDA ( **a**) and kNN ( **b**) classifiers.

Note that all the accuracy plots refer to the same dynamic classification problem (high arousal vs low arousal), performed using different classifiers (SVM, LDA, kNN) and features on different groups. Spectral: Ac_Im ( Figure 6), Ac_So ( Figure 7), Ps_Im ( Figure 8) and Ps_So ( Figure 9); temporal: Ac_So ( Figure 10), Ps_Im ( Figure 11) and Ps_So ( Figure 12).

Using spectral features, in all the groups some classifiers showed an accuracy greater than the benchmark. In the Ac_Im group, *ACC _LDA_* = 51.97% @ *t* = 0.080 *s* (t(18)=6.291, p<0.001) and *ACC _SVM_* = 51.07% @ *t* = 0.416 *s* (t(18)=6.531, p<0.001). In the Ac_So group, *ACC _LDA_* = 53.04% @ *t* = 0.332 *s* (t(18)=8.583, p<0.001) and *ACC _SVM_* = 51.16% @ *t* = 0.146 *s* (t(18)=8.612, p<0.001). In the Ps_Im group, *ACC _LDA_* = 53.12% @ *t* = 0.156 *s* (t(18)=6.372, p=0.000) and *ACC _SVM_* = 51.83% @ *t* = 0.140 *s* (t(18)=6.668, p<0.001). In the Ps_So group, *ACC _SVM_* = 50.62% @ *t* = 0.024 *s* (t(18)=5.236, p=0.003) and *ACC _kNN_* = 51.41% @ *t* = 0.476 *s* (t(18)=4.307, p=0.026).

Using temporal features, in only three groups did some classifiers show an accuracy greater than the benchmark. In the Ac_So group, *ACC _SVM_* = 63.80% @ *t* = 0.100 *s* (t(18)=6.113, p=0.001). In the Ps_Im group, *ACC _LDA_* = 63.68% @ *t* = 0.024 *s* (t(18)=12.108, p<0.001) and *ACC _SVM_* = 51.43% @ *t* = 0.084 *s* (t(18)=4.881, p=0.008). In the Ps_So group, *ACC _LDA_* = 64.30% @ *t* = 0.0276 *s* (t(18)=11.092, p<0.001) and *ACC _kNN_* = 63.70% @ *t* = 0.480 *s* (t(18)=16.621, p<0.001).

Table 4 reports the accuracies for dynamic features, ordered in descending order and grouped for classifier, feature group and time.

**Table 4.** Dynamic features. Ordered accuracies grouped for classifier, feature and group.

Classifier	Accuracy	Time [s]	Group	Feature
SVM	63.80%	0.1	Ac_So	Temporal
kNN	63.70%	0.048	Ps_So	Temporal
LDA	63.68%	0.024	Ps_Im	Temporal
LDA	63.30%	0.0276	Ps_So	Temporal
LDA	53.12%	0.156	Ps_Im	Spectral
LDA	53.04%	0.3332	Ac_So	Spectral
LDA	51.97%	0.08	Ac_Im	Spectral
SVM	51.83%	0.14	Ps_Im	Spectral
SVM	51.43%	0.084	Ps_Im	Temporal
kNN	51.41%	0.476	Ps_So	Spectral
SVM	51.16%	0.146	Ac_So	Spectral
SVM	51.07%	0.416	Ac_Im	Spectral
SVM	50.62%	0.024	Ps_So	Spectral

SVM, support vector machine; LDA, linear discriminant analysis; kNN, k-nearest neighbour.

## Discussion

The aim of the study was to provide new methodological insights regarding machine learning approaches for the classification of anticipatory emotion-related EEG signals, by testing the performance of different classifiers on different features.

From the ISIs (i.e. the 1000 ms long window preceding each stimulus onset), we extracted two kinds of “static” features, namely spectral and temporal, the most commonly used features in the field of emotion recognition ^19, 20^. As spectral features, we used the beta-over-alpha and the beta-over-theta ratio, whereas for the temporal feature we concatenated the decimated EEG values.

Additionally, we extracted the temporal sequences of both static spectral and temporal features, using a 500 ms long window moving along the ISI to build dynamic spectral and temporal features, respectively. This step is crucial for our work since, considering the temporal resolution of the EEG, an efficient classification should take into account the temporal dimension, to provide information about when the difference between two conditions are maximally expressed and therefore classified.

We trained and tested three different classifiers (LDA, SVM, kNN, the most commonly used in the field of emotion recognition ^19, 20^) using both static and dynamic features, comparing their accuracies against a random classifier that served as benchmark.

Our goal was to identify the best classifier (static vs dynamic) and the best feature type (spectral vs temporal) to classify the arousal level (high vs low) of 56 auditory/visual stimuli. The stimuli, extracted from two standardized datasets (NIMSTIM ^52^ and IADS ^44^), for visual and auditory stimuli, respectively) were presented in a randomized order, triggered by a TrueRNG™ hardware random number generator.

Considering the number of groups (four), the number of classifiers (three) and the number of feature types (two), each classification (static or dynamic) produced a total of 24 accuracies, whose significances were statistically tested (using a two-sample t-test and the benchmark’s accuracies).

Within the nine significant accuracies obtained using static features, the classifier that obtained the highest number of accuracies was the SVM (six significant accuracies), followed by kNN (two significant accuracies) and LDA (one significant accuracy). The most frequent feature was the temporal (five significant accuracies). Finally, the best (static) feature-classifier combination was the SVM with spectral features (51.8%), followed by LDA with spectral features (51.4%) and kNN with temporal features (51%).

Within the 13 significant accuracies obtained using dynamic features, the classifier that obtained the highest number of accuracies was the SVM (six significant accuracies), followed by LDA (four significant accuracies) and kNN (three significant accuracies). The most frequent feature was the spectral (eight significant accuracies). Finally, the best (dynamic) feature-classifier combination was the SVM with temporal features (63.8%), followed by kNN with temporal features (63.70%) and LDA with temporal features (63.68%). Spectral features produced only the 5th highest accuracy (53.12% with LDA). The three best accuracies were all within the first 100ms of the ISI, although a non-significant Spearman’s correlation between accuracy and time was observed (r=-0.308, p=0.306).

Our results show that globally the SVM presents the best accuracy, independent from feature type (temporal or spectral), but more importantly, the combination of SVM with the dynamic temporal feature produced the best classification performance. This finding is particularly relevant, considering the application of EEG in cognitive science. In fact, due to its high temporal resolution, EEG is often applied to investigate the timing of neural processes in relation to behavioural performance.

Our results therefore suggest that, in order to best classify emotions based on electrophysiological brain activity, the temporal dynamic of the EEG signal should be taken into account with a dynamic feature and consequently with a dynamic classifier. In fact, by including also time evolution of the feature in the machine learning model, it is possible to infer when two different conditions maximally diverge, allowing possible interpretation of the timing of the cognitive processes and the behaviour of the underlying neural substrate.

Finally, the main contribution of our results for the scientific community is that they provide a methodological advancement that is generally valid both for the investigation of emotion based on a machine learning approach with EEG signals and also for the investigation of preparatory brain activity.

## Data availability

### Underlying data

Figshare: EEG anticipation of random high and low arousal faces and sounds. https://doi.org/10.6084/m9.figshare.6874871.v8 ^27^

This project contains the following underlying data:

- EEG metafile (DOCX)
- EEG data related to the Passive, Active and Predictive conditions (CSV)
- Video clips of the EEG activity before stimulus presentation (MPG)

### Extended data

Figshare: EEG anticipation of random high and low arousal faces and sounds. https://doi.org/10.6084/m9.figshare.6874871.v8 ^27^

This project contains the following extended data:

- Detailed description of LDA, SVM and kNN machine learning algorithms (DOCX)

Data are available under the terms of the Creative Commons Attribution 4.0 International license (CC-BY 4.0).

## Software availability

Source code available from: https://github.com/mbilucaglia/ML_BAA

Archived source code at time of publication: https://doi.org/10.5281/zenodo.3666045 ^51^

License: GPL-3.0
