# Applying machine learning EEG signal classification to emotion‑related brain anticipatory activity

**DOI:** 10.12688/f1000research.22202.2

**Published:** 2021-10-08

**Authors:** Marco Bilucaglia, Gian Marco Duma, Giovanni Mento, Luca Semenzato, Patrizio E. Tressoldi

**Affiliations:** 1 Behavior and BrainLab, IULM, Milan, Italy; 2 Department of Developmental and Social Psychology (DPSS), Università degli Studi di Padova, Padova, Italy; 3 Department of General Psychology, Università degli Studi di Padova, Padova, Italy; 4 Science of Consciousness Research Group, Studium Patavinum, Università degli Studi di Padova, Padova, Italy

**Keywords:** EEG, brain anticipatory activity, machine learning, emotion

## Abstract

Machine learning approaches have been fruitfully applied to several neurophysiological signal classification problems. Considering the relevance of emotion in human cognition and behaviour, an important application of machine learning has been found in the field of emotion identification based on neurophysiological activity. Nonetheless, there is high variability in results in the literature depending on the neuronal activity measurement, the signal features and the classifier type. The present work aims to provide new methodological insight into machine learning applied to emotion identification based on electrophysiological brain activity. For this reason, we analysed previously recorded EEG activity measured while emotional stimuli, high and low arousal (auditory and visual) were provided to a group of healthy participants. Our target signal to classify was the pre-stimulus onset brain activity. Classification performance of three different classifiers (LDA, SVM and kNN) was compared using both spectral and temporal features. Furthermore, we also contrasted the performance of static and dynamic (time evolving) approaches. The best static feature-classifier combination was the SVM with spectral features (51.8%), followed by LDA with spectral features (51.4%) and kNN with temporal features (51%). The best dynamic feature classifier combination was the SVM with temporal features (63.8%), followed by kNN with temporal features (63.70%) and LDA with temporal features (63.68%). The results show a clear increase in classification accuracy with temporal dynamic features.

## Introduction

In last decades, the vision of the brain has moved from a passive stimuli elaborator to an active reality builder. In other words, the brain is able to extract information from the environment, building up inner models of external reality. These models are used to optimize the behavioural outcome when reacting to upcoming stimuli ^1– 4^.

One of the main theoretical models assumes that the brain, in order to regulate body reaction, runs an internal model of the body in the world, as described by embodied simulation framework ^5^. A much-investigated hypothesis is that the brain functions as a Bayesian filter for incoming sensory input; that is, it activates a sort of prediction based on previous experiences about what to expect from the interaction with the social and natural environment, including emotion ^6^. In light of this, it is possible to consider emotions, not only as a reaction to the external world, but also as partially shaped by our internal representation of the environment, which help us to anticipate possible scenarios and therefore to regulate our behaviour.

The construction model of emotion ^7^ argues that the human being actively builds-up his/her emotions in relation to the everyday life and social context in which they are placed. We actively generate a familiar range of emotions in our reality, based on their usefulness and relevance in our environment. In this scenario, in a familiar context we are able to anticipate which emotions will be probably elicited, depending on our model. As a consequence, the study of the anticipation of/preparation for forthcoming stimuli may represent a precious window for understanding the individual internal model and emotion construction process, resulting in a better understanding of human behaviour.

A strategy to study preparatory activity could be related to the experimental paradigm in which cues are provided regarding the forthcoming stimuli, allowing the investigation of the brain activity dedicated to the elaboration of incoming stimuli ^8, 9^. A cue experiment to predict the emotional valence of the forthcoming stimuli showed that the brain’s anticipatory activation facilitates, for example, successful reappraisal via reduced anticipatory prefrontal cognitive elaboration and better integration of affective information in the paralimbic and subcortical systems ^10^. Furthermore, preparation for forthcoming emotional stimuli also has relevant implications for clinical psychological conditions, such as mood disorders or anxiety ^11, 12^.

Recently, the study of brain anticipatory activity has been extended to statistically unpredictable stimuli ^13– 15^, providing experimental hints of specific anticipatory activity before stimuli are randomly presented. Starting from the abovementioned studies, we focused on the extension of brain anticipatory activity to statistically unpredictable emotional stimuli.

According to the so-called dimensional model, emotion can be defined in terms of three different attributes (or dimensions): valence, arousal and dominance. Valence measures the positiveness (ranging from unpleasant to pleasant), arousal measures the activation level (ranging from boredom to frantic excitement) and dominance measures the controllability (i.e. the sense of control) ^16^.

Emotions can be estimated from various physiological signals ^17, 18^, such as via skin conductance, electrocardiogram (ECG) and electroencephalogram (EEG). The latter has received a considerable amount of attention in the last decade, introducing several machine learning and signal processing techniques, originally developed in other contexts, such as text mining ^19^, data processing ^20^ and brain computer interfaces ^21, 22^. Emotion recognition has been re-drawn as a machine learning problem, where proper EEG related features are used as inputs to specific classifiers.

### State of the art

According to the literature, the most common features belong the spectral domain, in the form of spectral powers in delta, theta, alpha and gamma bands ^23^, as well as power spectral density (PSD) bins ^24^. The remaining belong to the time domain, in the form of event-related de/synchronizations (ERD/ERS) and event-related potentials (ERP) ^23^, as well as shape related indices such as the Hjorth parameters and the fractal dimension ^24^.

The most commonly used classifier is the support vector machine (SVM) with the radial basis function (RBF) kernel, followed by the k-nearest neighbour (kNN) and the linear discriminant analysis (LDA). When compared with neural network (NN) based classfiers (e.g., CNN, MLP-BP, ANN), SVM, kNN and LDA are adopted, complexively, 79.3% (NN 3.17%) ^23^, 84% (NN 15.5%) ^24^, and 44.4% (NN 5.6%) ^25^ of the times. Finally, most of the classifiers are implemented as non-adaptive (i.e. static) ^23^, in contrast to the dynamic versions that take into account the temporal variability of the features ^26^.

The classification performances are very variable because of the different features and classifiers adopted. The following examples are taken from ^23^ - in particular, from the subset (17 out of 63) of reviewed papers that focused on arousal classification. Using an SVM (RBF kernel) and spectral features (e.g. short-time Fourier transform), Lin and colleagues obtained 94.4% accuracy (i.e. percentage of corrected classification) ^27^, while using similar spectral features (e.g. PSD) and classifier (SVM with no kernel), Koelstra and colleagues obtained an accuracy of 55.7% ^28^. Liu and Sourina obtained an accuracy of 76.5% using temporal features (e.g. fractal dimension) with an SVM (no kernel) ^29^, while Murugappan and Murugappan obtained a an accuracy of 63% using similar temporal features and an SVM with a polynomial kernel ^30^. Finally, Thammasan and collegues obtained an accuracy of 85.3% using spectral features (e.g. PSD), but with a kNN (with k=3) ^31^.

The purpose of the present work is to provide new methodological advancements on the machine learning classification of emotions, based on the brain anticipatory activity.

Our main research question can be summarised as: what is the best classifier/features combination for the classification of the emotions in the brain anticipatory activity framework? For this purpose, we compared the performances of the tree most common classifiers (namely LDA, SVM, kNN) trained using two types of EEG features (namely, spectral and temporal). In addition to their popularity, the classifiers were selected as representative of 3 different families, namely *parametric* (LDA), *discriminative* (SVM) and *non-parametric* (kNN) classifiers, as well as 2 additional families, namely *linear* (LDA) and *non-linear* (SVM, kNN) classifiers. Each classifier was also trained followed a dynamic approach, to take into account the temporal variability of the features.

The results provide useful insights regarding the best classifier features configuration to better discriminate emotion-related brain anticipatory activity.

Within the extended data of the present article ^32^ we included a document (titled *Machine Learning Introduction*) briefly describing the classification problem. We are aware that the treatment is far from being fully exhaustive, but we hope it will serve as a comprehensive and self contained starting point for novice readers. 

## Methods

### Ethical statement

The data of the present study were obtained in the experiment described in 33, which was approved by the Ethical Committee of the Department of General Psychology, University of Padova (No. 2278). Before taking part in the experiment, each subject gave his/her informed consent in writing after having read a description of the experiment. In line with department policies, this re-analysis of an original study approved by the ethics committee did not require new ethical approval.

### Stimuli and experimental paradigm

In the present study, we reanalysed the EEG data ^32^ of the experiment described in 33, applying a static and dynamic classification approach by using the three different classifiers and two different feature types.

A more detailed description of the experimental design is available in the original study. Here we describe only the main characteristics.

Two sensory categories of stimuli (i.e. visual and auditory), were extracted according to their arousal value from two standardized international archives. Visual stimuli consisted of pictures of 28 faces, 14 neutral faces and 14 fearful faces were extracted from the NIMSTIM archive ^34^, whereas auditory stimuli consisted of 28 sounds, and 14 low- and 14 high-arousal sounds were chosen from the International Affective Digitized Sounds (IADS) archive ^35^. The stimuli were labelled as low or high arousal if the corresponding mean arousal score was lower or higher than 5, respectively.

To all 28 adult healthy participants, two different experimental tasks, which were delivered in separate blocks were presented. Within each task, the stimuli were randomly presented and equally distributed according to either sensory category (faces or sounds) and arousal level (high or low). Full details of these tasks have been described previously in 33.

### EEG recording

During the entire experiment, the EEG signal was continuously recorded using a Geodesic high density EEG system (EGI GES-300) through a pre-cabled 128-channel HydroCel Geodesic Sensor Net (HCGSN-128) referenced to the vertex (CZ), with a sampling rate of 500 Hz. The impedance was kept below 60kΩ for each sensor. To reduce the presence of EOG artefacts, subjects were instructed to limit both eye blinks and eye movements, as much as possible.

### EEG preprocessing

The continuous EEG signal was off-line band-pass filtered (0.1–45Hz) using a Hamming windowed sinc finite impulse response (FIR) filter (order = 16500) and then downsampled at 250 Hz. The EEG was epoched starting from 200 ms before the cue onset and ending at the stimulus onset. The initial epochs were 1300 ms long from the cue onset, including 300 ms of cue/fixation cross presentation and 1000 ms of interstimulus interval (ISI).

All epochs were visually inspected to remove bad channels and rare artefacts. Artefact reduced data were then subjected to independent component analysis (ICA) ^35^. All independent components were visually inspected, and those that related to eye blinks, eye movements, and muscle artefacts, according to their morphology and scalp distribution, were discarded. The remaining components were back-projected to the original electrode space to obtain cleaner EEG epochs.

The remaining ICA-cleaned epochs that still contained excessive noise or drift (±100 μV at any electrode) were rejected and the removed bad channels were reconstructed. Data were then re-referenced to the common average reference (CAR) and the epochs were baseline corrected by subtracting the mean signal amplitude in the pre-stimulus interval. From the original 1300 ms long epochs, final epochs were obtained only from the 1000 ms long ISI.

### Spectral features for static classification

From each epoch and each channel *k*, the PSD was estimated by a Welch’s periodogram using 250 points long Hamming’s windows with 50% overlapping. PSD was first log transformed to compensate the skewness of power values ^36^, then the spectral bins corresponding to alpha, beta and theta bands – defined as 6~13 *Hz,* 13~30 *Hz* and 4~6 *Hz*, respectively ^37^– were summed together. Finally, alpha, beta and theta total powers were computed as:

$$\beta_{tot}^{k}=\sum_{i\in [13;30]}PSD^{k}(i)\;$$

$$\alpha_{tot}^{k}=\sum_{i\in [6;13]}PSD^{k}(i)\;(14)$$

$$\theta_{tot}^{k}=\sum_{i\in [4;6]}PSD^{k}(i)\;$$

As a measure of emotional arousal, we computed the ratio between beta and alpha total powers $\beta_{tot}^{k}/\alpha_{tot}^{k}\;\;\;$ ^38^, while to measure cognitive arousal, we computed the ratio between beta and theta total powers $\theta_{tot}^{k}/\beta_{tot}^{k}\;\;\;$ ^39^.

For each epoch, the feature (with a dimensionality of 256) was obtained, concatenating the beta-over-alpha and beta-over-theta ratio of all the channels:

$$v≐\left[\frac{\beta_{tot}^{1}}{\alpha_{tot}^{1}},\frac{\theta_{tot}^{1}}{\beta_{tot}^{1}},\frac{\beta_{tot}^{2}}{\alpha_{tot}^{2}},\frac{\theta_{tot}^{2}}{\beta_{tot}^{2}},...,\frac{\beta_{tot}^{128}}{\alpha_{tot}^{128}},\frac{\theta_{tot}^{128}}{\beta_{tot}^{128}}\right]\;(15)$$

### Temporal features for static classification

It has been previously shown that arousal level (high or low) can be estimated from the contingent negative variation potentials ^33^. The feature extraction procedure, therefore, follows the classical approach for event-related potentials ^40^. Each epoch from each channel was first band pass filtered (0.05~10 *Hz*) using a zero-phase 2 ^nd^ order Butterworth filter and decimated to a sample frequency of 20 *Hz*. EEG signal was thus normalized (i.e. z-scored) according to the temporal mean and the temporal standard deviation:

$$x_{i}(t_{k})=({\tilde{x}}_{i}(t_{k})-m_{i})/s_{i}$$

where ${\tilde{x}}_{i}(t_{k})$ is the raw signal from i-th channel at time point *t _k_*, and *m _i_* and *s _i_* are, respectively, the temporal mean and the temporal standard deviation of the i-th channel. For each epoch, the feature (with a dimensionality of 2560) was obtained, concatenating all normalized signal from each channel:

$$v≐\left[x_{1}(t_{1}),x_{1}(t_{2}),...,x_{1}(t_{20}),...,x_{128}(t_{1}),x_{128}(t_{2}),...,x_{128}(t_{20})\right]\;(16)$$

### Dynamic approach

Each epoch was partitioned into 125 temporal segments, 500 ms long and shifted by 1/250 s (one sample). Within each time segment, we extracted the spectral and temporal features for dynamic classification, following the same approaches described in *Spectral features for static classification* and *Temporall features for static classification* sub-sections, respectively. Temporal features for dynamic classification had a dimensionality of 1280, corresponding to 0.5 × 20 = 10 samples per channel. Spectral features for dynamic classification had the same dimensionality as their static counterparts (256), but the Welch’s periodogram was computed using a 16 points long Hamming’s window (zero-padded to 250 points) with 50% overlapping.

### Feature reduction and classification

The extracted features (for both the static and dynamic approaches) were grouped according to the stimulus type (sound or image) and the task (active or passive), in order to classify the group-related arousal level (high or low). A total of four binary classification problems (high arousal vs low arousal) were performed: active image (Ac_Im), active sound (Ac_So), passive image (Ps_Im) and passive sound (Ps_So). For each classification problem, the two classes were approximatively balanced, as shown in the following Table 1. We chose a binary classification since it is associated to lower computational costs than the multiclass alternatives, that are usually obtained by a cascade of binary classifications (see *Machine Learning Introduction* within the *Extended data* ^32^).

**Table 1.** Class distribution. Distribution of the two classes (High arousal and Low arousal) for each classification problem.

Classification problem	# High arousal (%)	#Low arousal (%)
Ac_Im	1294 (51%)	1243 (49%)
Ac_So	1223 (49%)	1270 (51%)
Ps_Im	1215 (48%)	1305 (52%)
Ps_So	1279 (49%)	1302 (51%)

Features for static classification were reduced by means of the biserial correlation coefficient *r* ^2^ with the threshold set at 90% of the total feature score. In order to identify the discriminative power of each EEG channel, a series of scalp plots (one for each feature type and each group) of the coefficients were drawn. Since each channel is associated with *N* > 1 features (as well as *N r* ^2^ coefficients), the coefficients (one coefficient for each channel) are calculated as a mean value. In other words, spectral and temporal features had two and 20 scalar features, respectively, for each EEG channel. To compute their scalp plots, we averaged 2 and 20 *r* ^2^ coefficients of each channel. To enhance the visualization of the plots, the coefficients were finally normalized to the total score and expressed as a percentage.

Each classification problem was addressed by the mean of three classifiers: LDA with pseudo-inverse covariance matrix; soft-margin SVM with penalty parameter *C* = 1 and RBF kernel; and kNN with Euclidean distance and k=1. Additionally, a random classifier, giving a uniform pseudo-random class (Pr{HA} = Pr{LA} = 0.5), served as a benchmark ^41^. Since we were interested in the overall correct classification without distinguishing between the different classes, the accuracy was selected as evaluation metric ^42^. As long as the classes are approximatively balanced, the accuracy can be considered a straightforward and robust metric to assess the classifier performance (see *ML Introduction* in the *Extended data* ^32^).

The accuracy of the classifiers was measured, repeating 10 times for a 10 fold cross validation scheme. The feature selection was computed within each cross validation step, to avoid overfitting and reduce biased results ^43^.

For each group (Ac_Im, Ac_So, Ps_Im, Ps_So) and each feature type (static spectral, static temporal), the classification produced a 10 × 4 matrix containing the mean accuracies (one for each of the 10-fold cross-validation repetitions) of each classifier.

Features for dynamic classification were reduced and classified similarly to the static ones. For each temporal segment, the associated features were reduced by means of the biserial correlation coefficient (threshold at 90%) and the classifiers (SVM, kNN, LDA and random) were evaluated using a 10-fold cross-validation scheme – repeated 10 times.

For each group, each feature type (dynamic spectral, dynamic temporal), each temporal segment and each classifier, the classification produced 10 sequences of mean accuracies ${\left\{ACC_{i}\right\}}_{i=1}^{125}$ – one for each repetition of the 10-fold cross-validation scheme.

### Data analysis

The syntax in MATLAB used for all analyses is available on GitHub along with the instructions on how to use it (see *Software availability*) ^44^. The software can also be used with the open-source program Octave.

### Statistical analysis

The results of the static classifications were compared against the benchmark classifier by means of a two-sample t-test (right tail).

The results of dynamic classifications were compared following a segment-by-segment approach. For each group, the accuracy sequences of the dynamic classifiers (SVM, kNN and LDA) were compared with the benchmark accuracy sequence. Each sample $ACC_{i}^{k}$ , with *k* = {SVM, kNN, LDA}, was tested against $ACC_{i}^{\text{Random}}$ by means of two-sample t-tests (right tail). The corresponding p-value sequences ${\left\{p_{i}^{k}\right\}}_{i=1}^{125}$ were Bonferroni-Holm corrected for multiple comparisons. Finally, the best accuracy point was detected as the left extreme of the temporal window corresponding to the highest significant accuracy.

## Results

### Static approach

**Figure 1.**
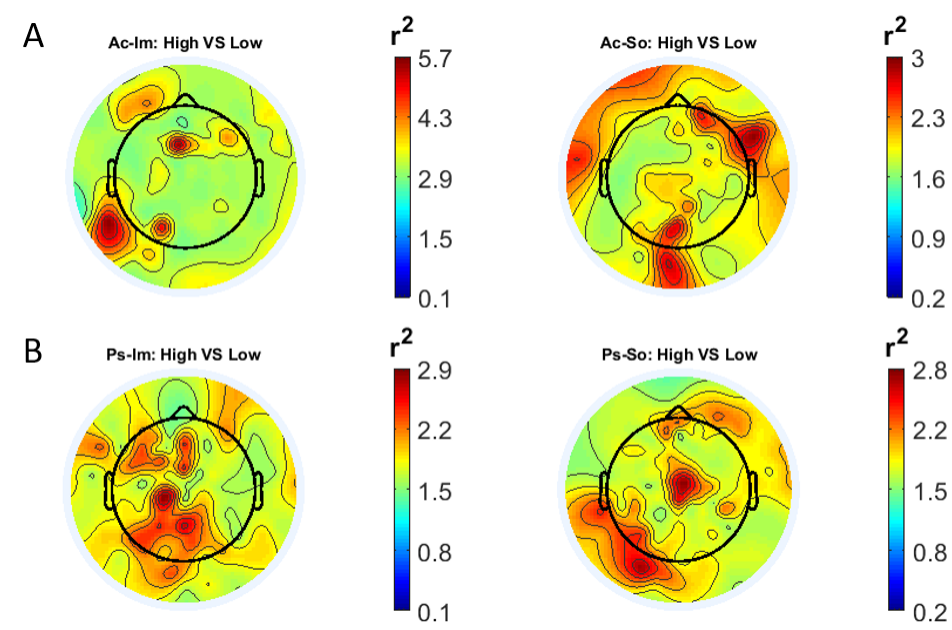
Spectral features. Scalp distribution of the *r* ^2^ coefficients (normalized to the total score and expressed as percentage) grouped for tasks and stimulus type. ( **a**) Active task: left Image, right Sound; ( **b**) Passive task: left Image, right Sound.

**Figure 2.**
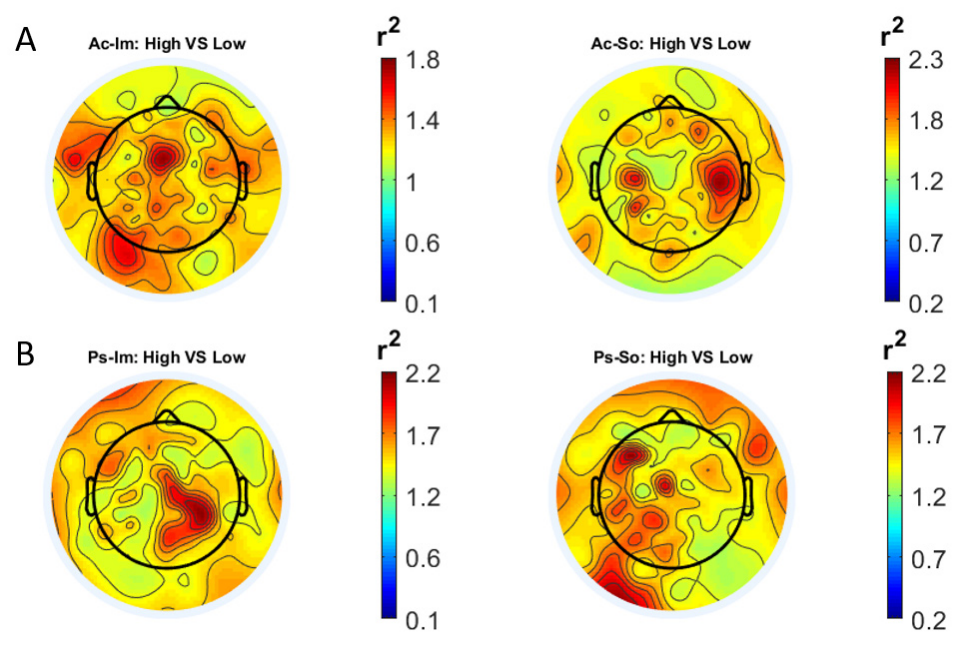
Temporal features. Scalp distribution of the *r* ^2^ coefficients (normalized to the total score and expressed as percentage), grouped for tasks and stimulus type. ( **a**) Active task: left Image, right Sound; ( **b**) Passive task: left Image, right Sound.

In Figure 1 and Figure 2, the scalp distributions of *r* ^2^ coefficients for each binary static classification problem, grouped for feature (spectral, temporal) and groups (Ps_Im, Ps_So, Ac_Im, Ac_So), are shown.

The temporal feature gave the most consistent topographical pattern, showing that the regions that best differentiate between high vs low stimuli (auditory and visual) were located over the central-parietal electrodes, whereas a more diffuse pattern in the scalp topography emerged for the spectral features.

**Figure 3.**
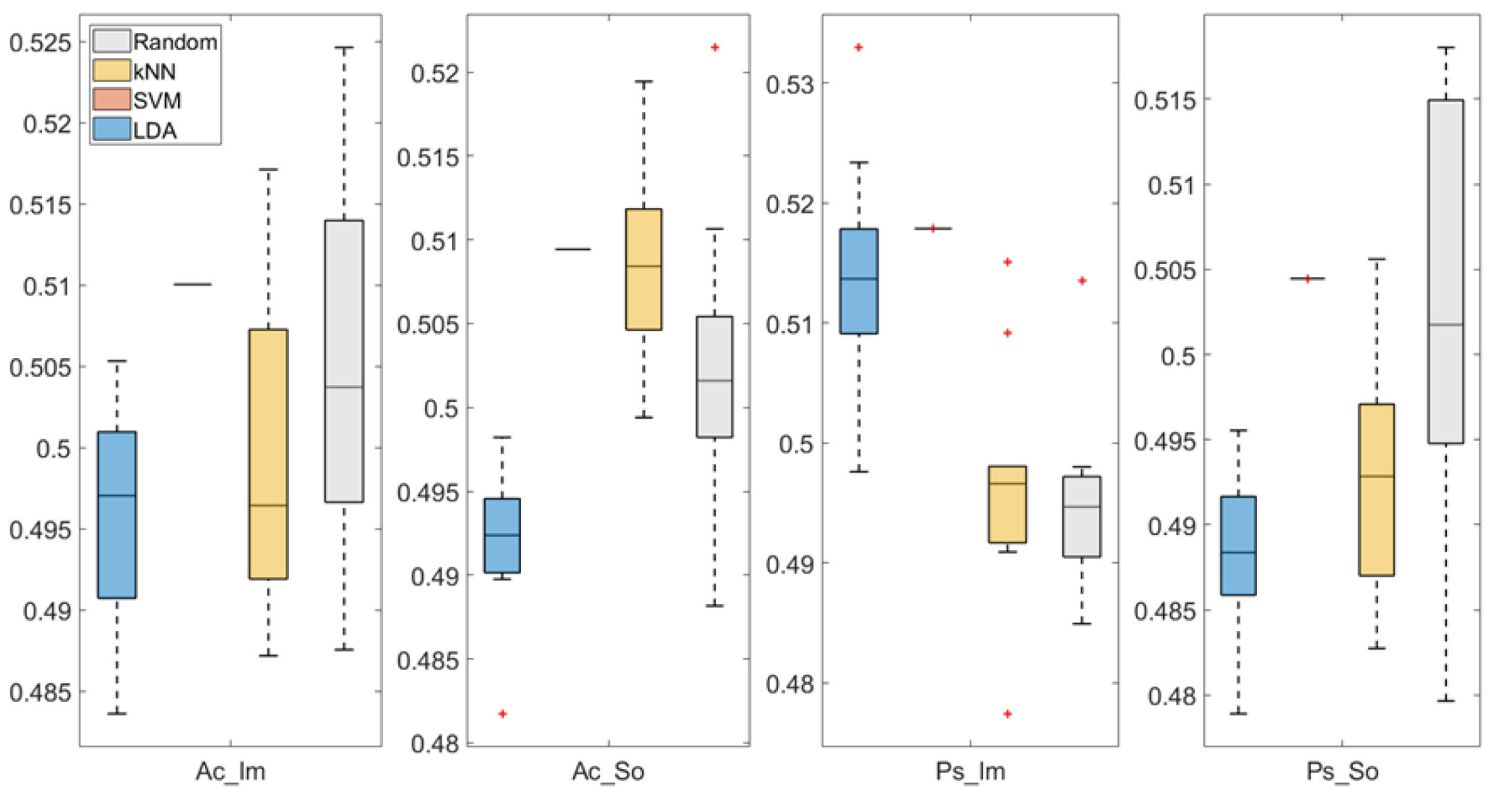
Box-plots of the accuracies of the static spectral classifications. From left: Active Image (Ac_Im), Active Sound (Ac_So), Passive Image (Ps_Im) and Passive Sound (Ps_So).

**Figure 4.**
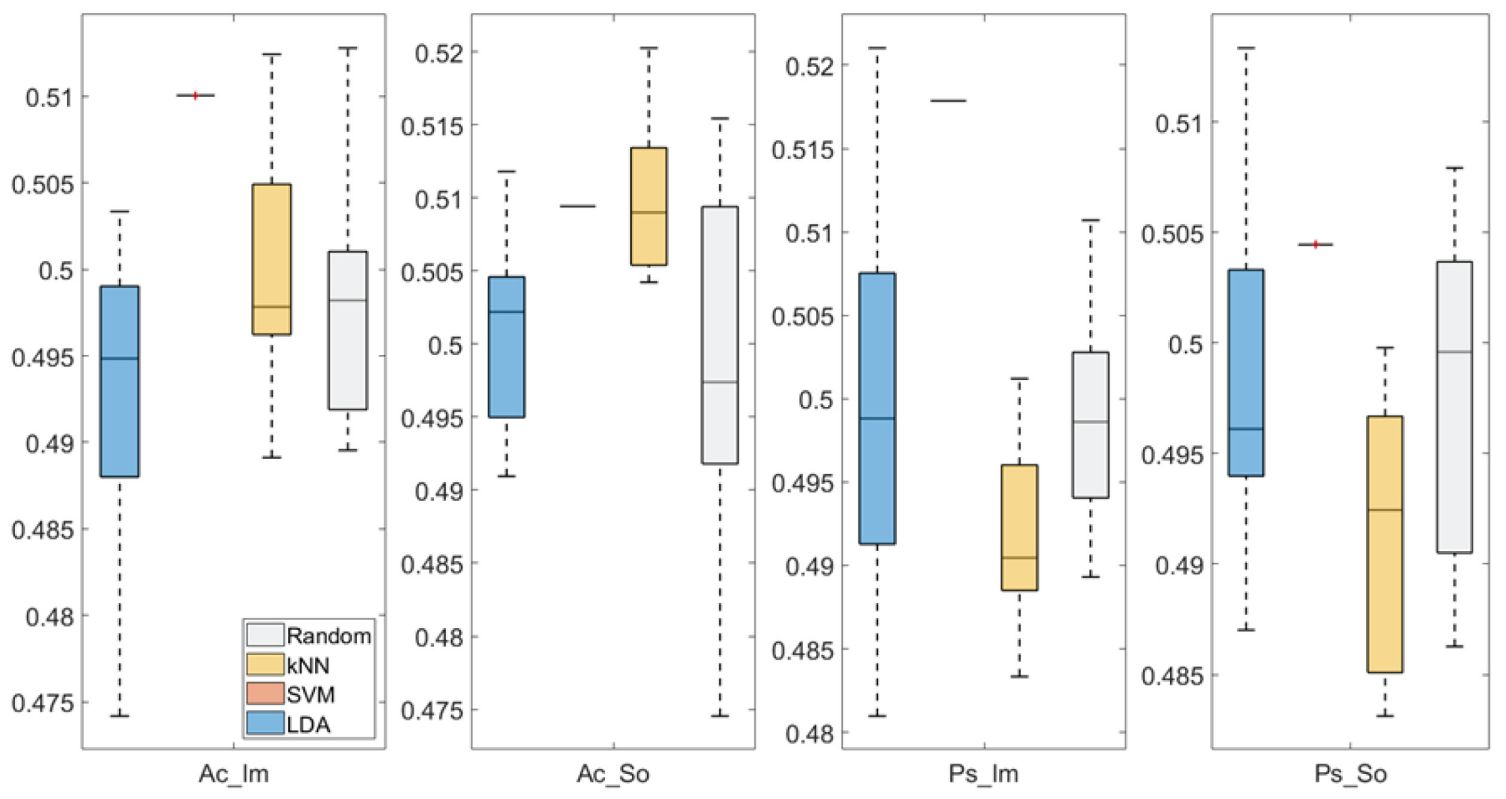
Box-plots of the accuracies of the static temporal classifications. From left: Active Image (Ac_Im), Active Sound (Ac_So), Passive Image (Ps_Im) and Passive Sound (Ps_So).

In Figure 3 and Figure 4, box plots of the accuracies of static temporal and spectral classifications, grouped for condition, are shown. Note that SVM accuracies (the 2 ^nd^ boxplot from the left) are always shown as lines because the accuracies were constant within each cross-validation step (see also Table 2, Table 3 and Table 4).

**Table 2.** Static features. Ordered accuracies grouped for classifier, feature and group.

Classifier	Accuracy	Feature	Group
SVM	51.80%	Spectral	Ps_Im
LDA	51.40%	Spectral	Ps_Im
kNN	51%	Temporal	Ac_So
kNN	50.90%	Spectral	Ac_So
SVM	50.90%	Spectral	Ac_So
SVM	50.90%	Temporal	Ac_So
SVM	50.40%	Temporal	Ps_So

SVM, support vector machine; LDA, linear discriminant analysis; kNN, k-nearest neighbour.

**Table 3.** Mean (M) and standard deviations (SD) of the accuracies of the static spectral classifications. Active Image (Ac_Im), Active Sound (Ac_So), Passive Image (Ps_Im) and Passive Sound (Ps_So).

Group	LDA	SVM	kNN	Random
Ac_Im	M=0.496, SD=0.007	M=0.510, SD=0.000	M=0.500, SD=0.010	M=0.505, SD=0.011
Ac_So	M=0.492, SD=0.004	M=0.509, SD=0.000	M=0.509, SD=0.007	M=0.503, SD=0.009
Ps_Im	M=0.514, SD=0.010	M=0.518, SD=0.000	M=0.496, SD=0.010	M=0.495, SD=0.008
Ps_So	M=0.488, SD=0.005	M=0.504, SD=0.000	M=0.493, SD=0.007	M=0.503, SD=0.013

SVM, support vector machine; LDA, linear discriminant analysis; kNN, k-nearest neighbour.

**Table 4.** Mean (M) and standard deviations (SD) of the accuracies of the static temporal classifications. Active Image (Ac_Im), Active Sound (Ac_So), Passive Image (Ps_Im) and Passive Sound (Ps_So).

Group	LDA	SVM	kNN	Random
Ac_Im	M=0.492, SD=0.010	M=0.510, SD=0.000	M=0.500, SD=0.008	M=0.498, SD=0.007
Ac_So	M=0.501, SD=0.007	M=0.509, SD=0.000	M=0.510, SD=0.006	M=0.498, SD=0.012
Ps_Im	M=0.500, SD=0.012	M=0.518, SD=0.000	M=0.492, SD=0.005	M=0.499, SD=0.006
Ps_So	M=0.499, SD=0.008	M=0.504, SD=0.000	M=0.492, SD=0.006	M=0.498, SD=0.008

SVM, support vector machine; LDA, linear discriminant analysis; kNN, k-nearest neighbour.

Note that all the accuracies refer to the same static classification problem (high arousal vs low arousal), performed using different classifiers (SVM, LDA, kNN) and features (spectral, temporal), on different groups (Ps_Im, Ps_So, Ac_Im, Ac_So).

Using spectral features, in only two groups some classifiers showed an accuracy greater than the benchmark. In the Ac_So group, *ACC _SVM_* = 50.9% (t(18)=2.371, p=0.015) and *ACC _kNN_* = 50.9% (t(18)=1.828, p=0.042), while for Ps_Im, *ACC _LDA_* = 51.4% (t(18)=4.667, p<0.001) and *ACC _SVM_* = 51.8% (t(18)=9.513, p<0.001).

Using temporal features, in all the groups some classifiers showed an accuracy greater than the benchmark. In the Ac_So group, *ACC _SVM_* = 50.9% (t(18)=2.907, p=0.005) and *ACC _kNN_* = 51% (t(18)=2.793, p=0.006) and in the Ps_So group, *AAC _SVM_* = 50.4% (t(18)=9.493, p<0.001).

### Dynamic approach

**Figure 5.**
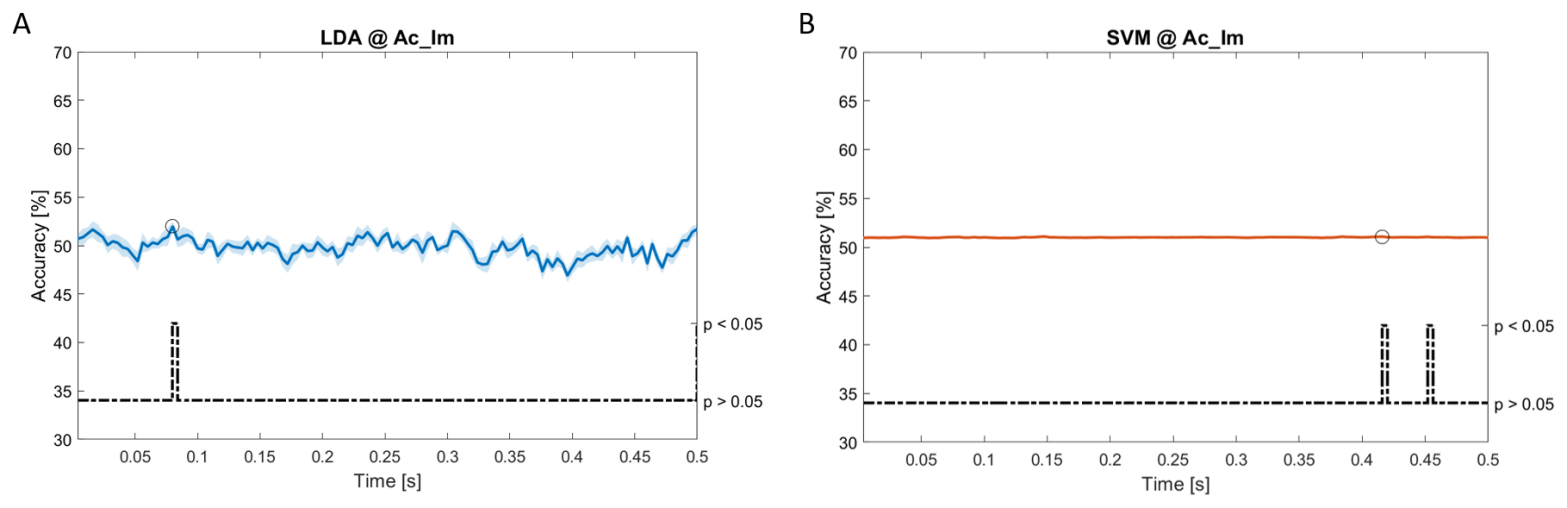
Spectral dynamic features. Accuracy (mean value, coloured line; standard deviation, shaded line) and p-values (black dotted line) in Ac_Im group for LDA ( **a**) and SVM ( **b**) classifiers.

In Figure 5– Figure 11, the results of the significant dynamic classifications are shown. In the upper section of the plots, the mean (bold line) and the standard deviation (shaded) of the accuracy sequence are shown. In the lower section of the plot (black dashed line), the Bonferroni-Holm corrected p-values sequence, discretized (as a stair graph) as significant (p<0.05) or non-significant (p>0.05) is shown.

**Figure 6.**
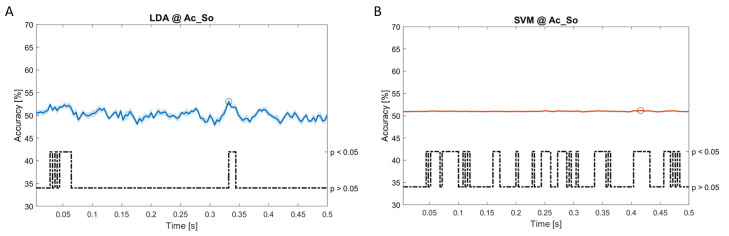
Spectral dynamic features. Accuracy (mean value, coloured line; standard deviation, shaded line) and p-values (black dotted line) in Ac_So group for LDA ( **a**) and SVM ( **b**) classifiers.

**Figure 7.**
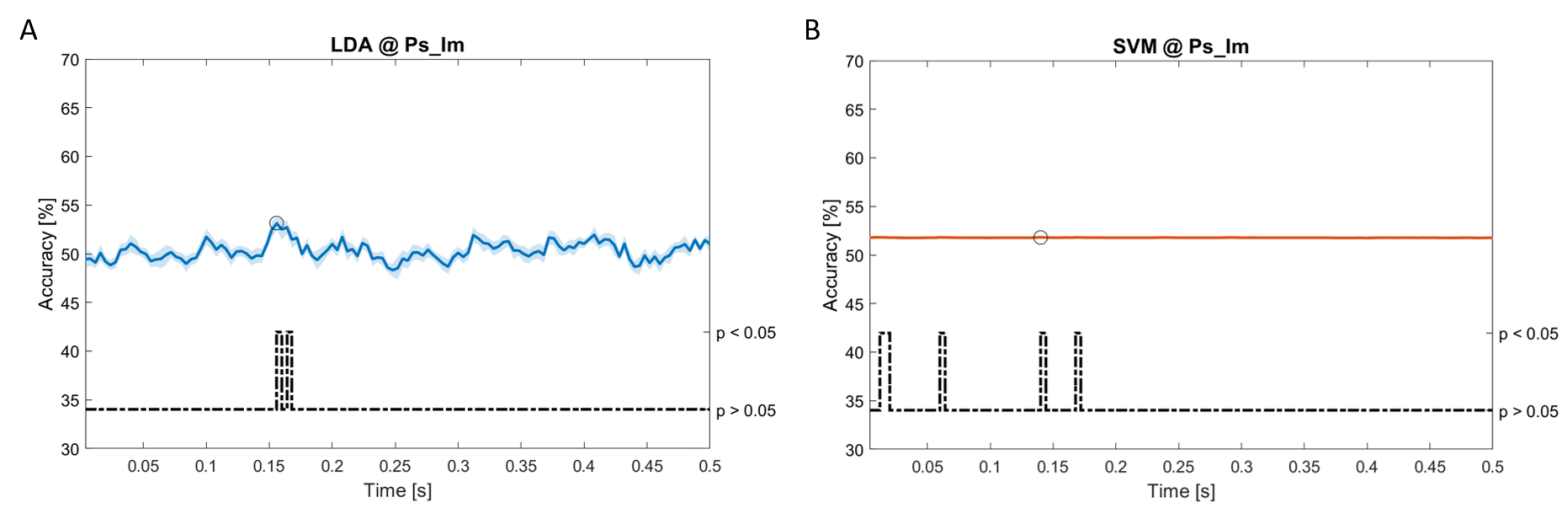
Spectral dynamic features. Accuracy (mean value, coloured line; standard deviation, shaded line) and p-values (black dotted line) in Ps_Im group for LDA ( **a**) and SVM ( **b**) classifiers.

**Figure 8.**
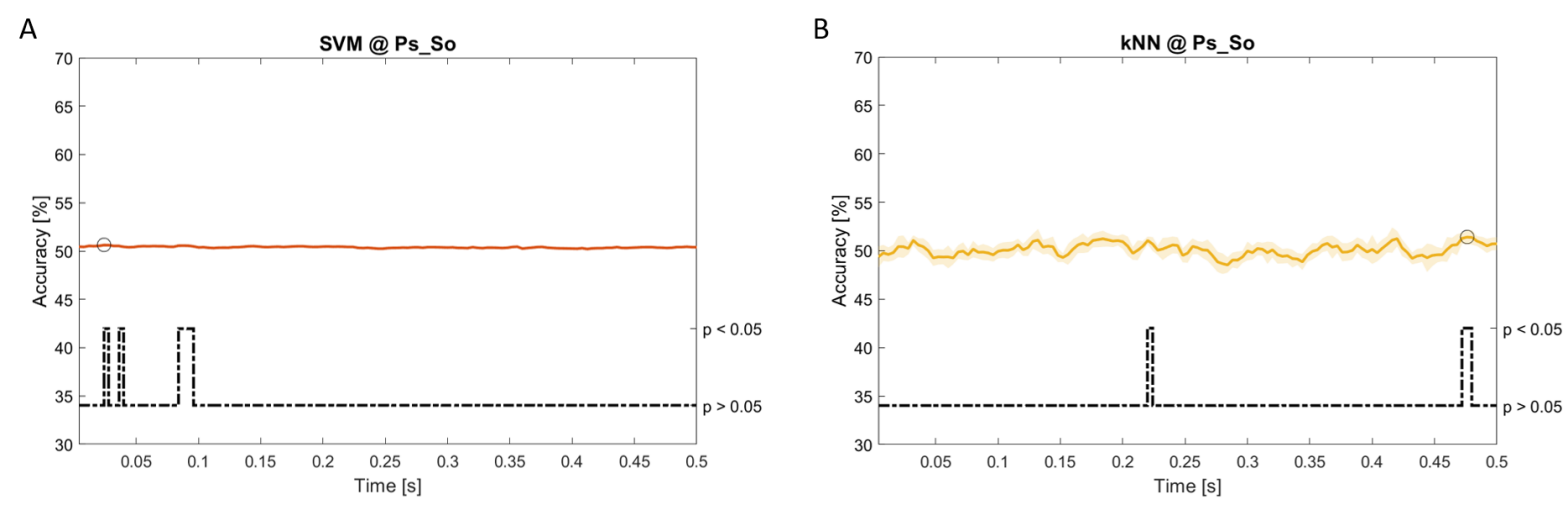
Spectral dynamic features. Accuracy (mean value, coloured line; standard deviation, shaded line) and p-values (black dotted line) in Ac_So group for SVM ( **a**) and kNN ( **b**) classifiers.

**Figure 9.**
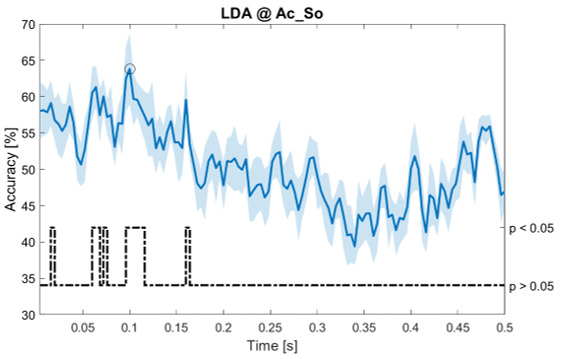
Temporal dynamic features. Accuracy (mean value, coloured line; standard deviation, shaded line) and p-values (black dotted line) in Ac_So group for LDA classifier.

**Figure 10.**
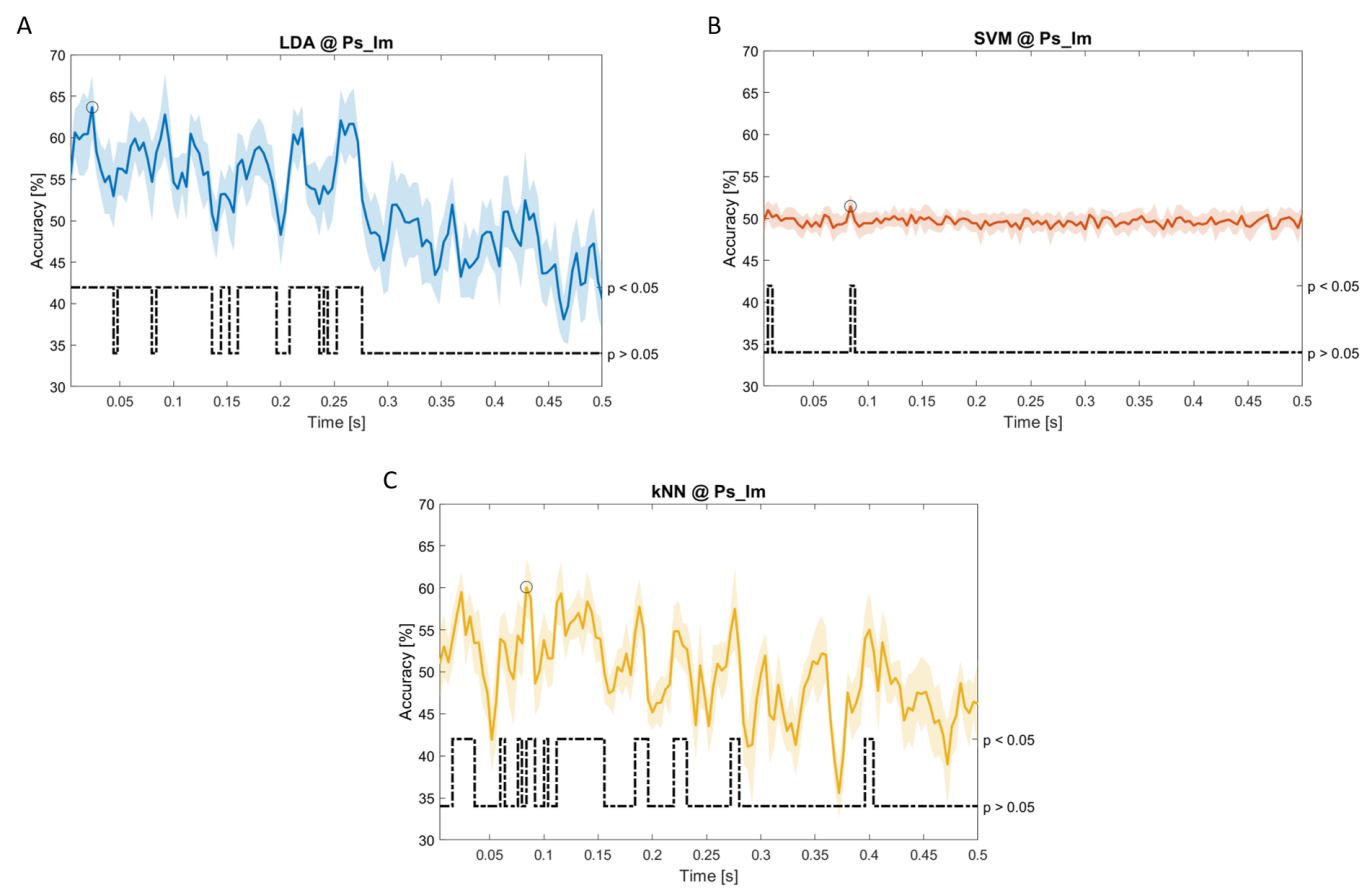
Temporal dynamic features. Accuracy (mean value, coloured line; standard deviation, shaded line) and p-values (black dotted line) in Ps_Im group for LDA ( **a**), SVM ( **b**) and kNN ( **c**) classifiers.

**Figure 11.**
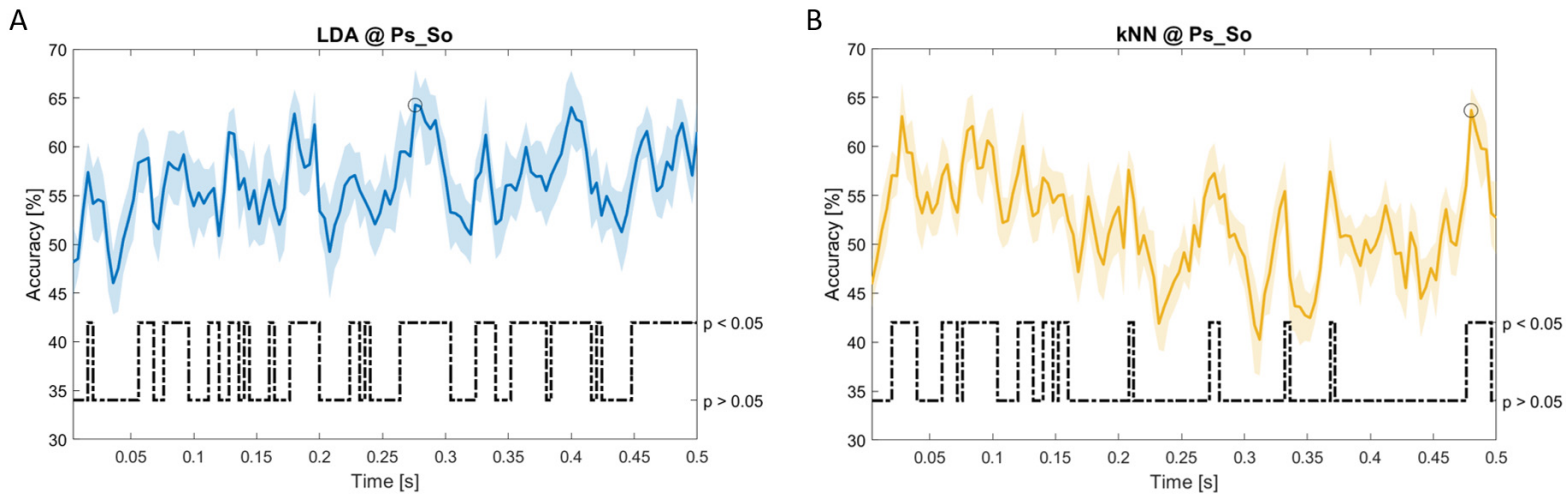
Temporal dynamic features. Accuracy (mean value, coloured line; standard deviation, shaded line) and p-values (black dotted line) in Ps_So group for LDA ( **a**) and kNN ( **b**) classifiers.

Note that all the accuracy plots refer to the same dynamic classification problem (high arousal vs low arousal), performed using different classifiers (SVM, LDA, kNN) and features on different groups. Spectral: Ac_Im ( Figure 5), Ac_So ( Figure 6), Ps_Im ( Figure 7) and Ps_So ( Figure 8); temporal: Ac_So ( Figure 9), Ps_Im ( Figure 10) and Ps_So ( Figure 11).

Using spectral features, in all the groups some classifiers showed an accuracy greater than the benchmark. In the Ac_Im group, *ACC _LDA_* = 51.97% @ *t* = 0.080 *s* (t(18)=6.291, p<0.001) and *ACC _SVM_* = 51.07% @ *t* = 0.416 *s* (t(18)=6.531, p<0.001). In the Ac_So group, *ACC _LDA_* = 53.04% @ *t* = 0.332 *s* (t(18)=8.583, p<0.001) and *ACC _SVM_* = 51.16% @ *t* = 0.146 *s* (t(18)=8.612, p<0.001). In the Ps_Im group, *ACC _LDA_* = 53.12% @ *t* = 0.156 *s* (t(18)=6.372, p=0.000) and *ACC _SVM_* = 51.83% @ *t* = 0.140 *s* (t(18)=6.668, p<0.001). In the Ps_So group, *ACC _SVM_* = 50.62% @ *t* = 0.024 *s* (t(18)=5.236, p=0.003) and *ACC _kNN_* = 51.41% @ *t* = 0.476 *s* (t(18)=4.307, p=0.026).

Using temporal features, in only three groups did some classifiers show an accuracy greater than the benchmark. In the Ac_So group, *ACC _SVM_* = 63.80% @ *t* = 0.100 *s* (t(18)=6.113, p=0.001). In the Ps_Im group, *ACC _LDA_* = 63.68% @ *t* = 0.024 *s* (t(18)=12.108, p<0.001) and *ACC _SVM_* = 51.43% @ *t* = 0.084 *s* (t(18)=4.881, p=0.008). In the Ps_So group, *ACC _LDA_* = 64.30% @ *t* = 0.0276 *s* (t(18)=11.092, p<0.001) and *ACC _kNN_* = 63.70% @ *t* = 0.480 *s* (t(18)=16.621, p<0.001).

Table 5 reports the accuracies for dynamic features, ordered in descending order and grouped for classifier, feature group and time.

**Table 5.** Dynamic features. Ordered accuracies grouped for classifier, feature and group.

Classifier	Accuracy	Time [s]	Group	Feature
SVM	63.80%	0.1	Ac_So	Temporal
kNN	63.70%	0.048	Ps_So	Temporal
LDA	63.68%	0.024	Ps_Im	Temporal
LDA	63.30%	0.0276	Ps_So	Temporal
LDA	53.12%	0.156	Ps_Im	Spectral
LDA	53.04%	0.3332	Ac_So	Spectral
LDA	51.97%	0.08	Ac_Im	Spectral
SVM	51.83%	0.14	Ps_Im	Spectral
SVM	51.43%	0.084	Ps_Im	Temporal
kNN	51.41%	0.476	Ps_So	Spectral
SVM	51.16%	0.146	Ac_So	Spectral
SVM	51.07%	0.416	Ac_Im	Spectral
SVM	50.62%	0.024	Ps_So	Spectral

SVM, support vector machine; LDA, linear discriminant analysis; kNN, k-nearest neighbour.

## Discussion

The aim of the study was to provide new methodological insights regarding machine learning approaches for the classification of anticipatory emotion-related EEG signals, by testing the performance of different classifiers on different features.

From the ISIs (i.e. the 1000 ms long window preceding each stimulus onset), we extracted two kinds of “static” features, namely spectral and temporal, the most commonly used features in the field of emotion recognition ^23, 24^. As spectral features, we used the beta-over-alpha and the beta-over-theta ratio, whereas for the temporal feature we concatenated the decimated EEG values.

Additionally, we extracted the temporal sequences of both static spectral and temporal features, using a 500 ms long window moving along the ISI to build dynamic spectral and temporal features, respectively. This step is crucial for our work since, considering the temporal resolution of the EEG, an efficient classification should take into account the temporal dimension, to provide information about when the difference between two conditions are maximally expressed and therefore classified.

We trained and tested three different classifiers (LDA, SVM, kNN, the most commonly used in the field of emotion recognition ^23, 24^) following both static and dynamic approaches, comparing their accuracies against a random classifier that served as benchmark.

Our goal was to identify the best combination of approach (static vs dynamic), classifier (LDA vs SVM vs kNN) and feature (spectral vs temporal) to classify the arousal level (high vs low) of 56 auditory/visual stimuli. The stimuli, extracted from two standardized datasets (NIMSTIM ^45^ and IADS ^34^), for visual and auditory stimuli, respectively) were presented in a randomized order, triggered by a TrueRNG™ hardware random number generator.

Considering the number of groups (four), the number of classifiers (three) and the number of feature types (two), each classification (static or dynamic) produced a total of 24 accuracies, whose significances were statistically tested (using a two-sample t-test and the benchmark’s accuracies).

Within the nine significant accuracies obtained using a static approach, the classifier that obtained the highest number of accuracies was the SVM (six significant accuracies), followed by kNN (two significant accuracies) and LDA (one significant accuracy). The most frequent feature was the temporal (five significant accuracies). Finally, the best (static) feature-classifier combination was the SVM with spectral features (51.8%), followed by LDA with spectral features (51.4%) and kNN with temporal features (51%).

Within the 13 significant accuracies obtained using a dynamic approach, the classifier that obtained the highest number of accuracies was the SVM (six significant accuracies), followed by LDA (four significant accuracies) and kNN (three significant accuracies). The most frequent feature was the spectral (eight significant accuracies). Finally, the best (dynamic) feature-classifier combination was the SVM with temporal features (63.8%), followed by kNN with temporal features (63.70%) and LDA with temporal features (63.68%). Spectral features produced only the 5th highest accuracy (53.12% with LDA). The three best accuracies were all within the first 100ms of the ISI, although a non-significant Spearman’s correlation between accuracy and time was observed (r=-0.308, p=0.306).

Table 6, summarises the three best (in terms of accuracy) classifier/features combinations for both the static and dynamic approaches.

**Table 6.** Best classifier/features combinations for static and dynamic approach.

Static classification			Dynamic classification		
Classifier	Classifier	Classifier	Classifier	Features	Accuracy
SVM	SVM	SVM	SVM	Temporal	63.87%
LDA	LDA	LDA	kNN	Temporal	63.70%
kNN	kNN	kNN	LDA	Temporal	53.12%

Our results show that globally the SVM presents the best accuracy, independent from feature type (temporal or spectral). This is in line with previous studies were SVM outperformed other classifiers such as NN and Random Forests ^46^. More importantly, the combination of SVM with the dynamic temporal feature produced the best classification performance. This finding is particularly relevant, considering the application of EEG in cognitive science. In fact, due to its high temporal resolution, EEG is often applied to investigate the timing of neural processes in relation to behavioural performance.

Our results therefore suggest that, in order to best classify emotions based on electrophysiological brain activity, the temporal dynamic of the EEG signal should be taken into account with a dynamic classifier. In fact, by including also time evolution of the feature in the machine learning model, it is possible to infer when two different conditions maximally diverge, allowing possible interpretation of the timing of the cognitive processes and the behaviour of the underlying neural substrate.

Finally, the main contribution of our results for the scientific community is that they provide a methodological advancement that is generally valid both for the investigation of emotion based on a machine learning approach with EEG signals and also for the investigation of preparatory brain activity.

### Study limitations

Nevertheless, the present study presents some limitations.

Despite being comparable with previous studies, the obtained accuracy is lower than those obtained with more complex classifiers, such as those based on Convolutional Neural Networks (CNNs). For example, feeding temporal features into a CNN classifier, some authors reported accuracies up to 86.5% ^47^, while others reported accuracies up 98.9% by training a CNN classifier with spectral features ^48^.

Additionally, the evaluation of the proposed classifiers is based on the accuracy, that can be still considered robust because of the class balance within each dataset. However, by considering other metrics (such as the MCC, the F-score, the Cohen’s kappa) or analysing the confusion matrices, the misclassifications could be more deeply analysed and, therefore, the classifiers could be more effectively tuned to an optimal point.

Finally, the discretization of the stimuli into two classes only (low and high) instead of multiple ones (e.g., low, medium, high) lowered the training/test computational costs but could represent a sub-optimal solution in terms of classification accuracies. By adding multiple classes, that is by discretizing the stimuli into finer arousal levels, the accuracy could be boosted, at the cost of lowering the number of available instances per class and increasing the chance of overfitting the models.

## Data availability

### Underlying data

Figshare: EEG anticipation of random high and low arousal faces and sounds. https://doi.org/10.6084/m9.figshare.6874871.v8 ^32^

This project contains the following underlying data:

- EEG metafile (DOCX)
- EEG data related to the Passive, Active and Predictive conditions (CSV)
- Video clips of the EEG activity before stimulus presentation (MPG)

### Extended data

Figshare: EEG anticipation of random high and low arousal faces and sounds. https://doi.org/10.6084/m9.figshare.6874871.v8 ^32^

This project contains the following extended data:

- Detailed description of LDA, SVM and kNN machine learning algorithms (DOCX)

Data are available under the terms of the Creative Commons Attribution 4.0 International license (CC-BY 4.0).

### Software availability

Source code available from: https://github.com/mbilucaglia/ML_BAA

Archived source code at time of publication: https://doi.org/10.5281/zenodo.3666045 ^44^

License: GPL-3.0
