# Supplementary material for: Applying machine learning EEG signal classification to emotion‑related brain anticipatory activity
Source: F1000Res. 2021 Oct 13;9:173. Originally published 2020 Mar 10. [Version 3] doi: 10.12688/f1000research.22202.3 (PMC10603316; doi:10.12688/f1000research.22202.3)
Supplement: Peer review history

# Reviewer report: Reviewer response for version 3

**DOI:** 10.5256/f1000research.78332.r210156

**Published:** 2023-10-26

**Authors:** Alessandro Bruno

**Affiliations:** 1 IULM University, Milan, Italy

"The manuscript tackles a meaningful and complex topic such as Neurophysiological signal analysis via Machine Learning. In particular, the authors focused on EEG for brain anticipatory activity signals, which bring in high-variance and noisy data. The latter raises a question concerning the reliability of machine learning techniques on the knowledge inference of the substantiated processes tracked by EEG. To this end, the manuscript tackles analysing the mentioned data using well-known machine learning techniques like SVM, LDA and kNN. Interestingly, the authors dived into a tricky problem: multimodal data analysis. They arranged experimental sessions with visual and auditory features. Participants were carefully instructed to keep eye movements and blinks as low as possible. Afterwards, FIR filters were adopted to extract a band-pass-filtered version of the signals, which underwent visual inspection to discard bad channels and rare artefacts. On top of that, ICA (Independent Component Analysis) is run to reduce data dimensions. 

 Then, the work presents two different approaches: static and dynamic. Both rely on spatial and temporal feature extraction, with the latter building upon Welch’s periodogram. A statistical significance test (Bonferroni-Holm) assessed the three classifiers (SVM, LDA, and kNN). 

 From an overall standpoint, the significant contributions of the article are three: a comparison between static and dynamic approaches over three traditional machine learning methods for brain anticipatory activity signal classification; experimental results from SVM, LDA, and kNN with heterogeneous data (temporal and spatial, auditory and visual), a comparison study in the discussion section with some SOTA (state-of-the-art) techniques. 

 I appreciated the outlooks drawn by the authors pointing out the current limitations in their method, especially when figures from Deep Learning models are compared with more "traditional machine learning" SVM, kNN and LDA."

Is the rationale for developing the new method (or application) clearly explained?

Yes

Is the description of the method technically sound?

Yes

Are the conclusions about the method and its performance adequately supported by the findings presented in the article?

Yes

If any results are presented, are all the source data underlying the results available to ensure full reproducibility?

Yes

Are sufficient details provided to allow replication of the method development and its use by others?

Yes

Reviewer Expertise:

Computer Vision, Eye-tracking, Perception, Artificial Intelligence, Machine Learning, Deep Learning.

I confirm that I have read this submission and believe that I have an appropriate level of expertise to confirm that it is of an acceptable scientific standard.

# Reviewer report: Reviewer response for version 3

**DOI:** 10.5256/f1000research.78332.r96822

**Published:** 2021-10-28

**Authors:** Rajesh K Tripathy

**Affiliations:** 1 Department of Electrical and Electronics Engineering, BITS-Pilani, Hyderabad, Telangana, 500078, India

My comments are correctly addressed.

Is the rationale for developing the new method (or application) clearly explained?

Yes

Is the description of the method technically sound?

Yes

Are the conclusions about the method and its performance adequately supported by the findings presented in the article?

Yes

If any results are presented, are all the source data underlying the results available to ensure full reproducibility?

Yes

Are sufficient details provided to allow replication of the method development and its use by others?

Yes

Reviewer Expertise:

NA

I confirm that I have read this submission and believe that I have an appropriate level of expertise to confirm that it is of an acceptable scientific standard.

# Reviewer report: Reviewer response for version 1

**DOI:** 10.5256/f1000research.24487.r92735

**Published:** 2021-09-21

**Authors:** Rajesh K Tripathy

**Affiliations:** 1 Department of Electrical and Electronics Engineering, BITS-Pilani, Hyderabad, Telangana, 500078, India

In this paper, authors have applied machine learning to classify emotion‑related brain anticipatory activity using EEG signals. The paper has several flaws and it should be carefully revised before resubmission. My review comments are given as follows:

1. Why authors have considered linear discriminant analysis, support vector machine, and k-nearest neighbor classifiers for the classification of EEG signals? Why not RNNs and multilayer perceptron.
2. Could you please add the arousal score? How you have divided into low-arousal and high-arousal. Why not three classes as low arousal vs. medium arousal vs. high arousal. Please clarify.
3. The cohen kappa score, Mathews correlation coefficients, F score values need to be evaluated.
4. Recently, the rhythm-specific deep CNN is successful for the classification of emotions using EEG signals. Could you compare with CNN-based methods?
5. The advantages and disadvantages of your work must be written.

Is the rationale for developing the new method (or application) clearly explained?

Yes

Is the description of the method technically sound?

Yes

Are the conclusions about the method and its performance adequately supported by the findings presented in the article?

Yes

If any results are presented, are all the source data underlying the results available to ensure full reproducibility?

Yes

Are sufficient details provided to allow replication of the method development and its use by others?

Yes

Reviewer Expertise:

AI for healthcare, deep learning, Biomedical signal processing, machine learning

I confirm that I have read this submission and believe that I have an appropriate level of expertise to confirm that it is of an acceptable scientific standard, however I have significant reservations, as outlined above.

# Response

**Published:** 2021-10-04

**Authors:** Patrizio Tressoldi

**Affiliations:** Studium Patavinum - Padova University, Italy

1. Why authors have considered linear discriminant analysis, support vector machine, and k-nearest neighbor classifiers for the classification of EEG signals? Why not RNNs and multilayer perceptron.

**Reply: According to the literature (ref. 19-20), LDA, SVM and kNN are the most commonly used (79.3% and 84% overall, respectively) classifiers in the Emotion recognition Field. A more recent review (ref. 53) partially confirmed this, showing that LDA, SVM and kNN are overall used 44.44% of the times. In contrast, neural‑network-based classifiers (e.g. CNN, MLP-BP, ANN) are used only 3.17% (ref. 19), 15.5% (ref. 20) and 5.56% (ref. 53) of the times.** **In addition to their popularity, LDA SVM and kNN were selected as representative of 3 different families, namely parametric, discriminative and non-parametric classifiers.**

1. Could you please add the arousal score? How you have divided into low-arousal and high-arousal. Why not three classes as low arousal vs. medium arousal vs. high arousal. Please clarify.

**Reply: Both the image and sounds were discretized into low and high arousal as those with a SAM score respectively lower and higher than 5.** **We chose a binary classification since it is associated to lower computational costs than the multiclass alternatives. Multiclass classification could produce different (and probably better) results, at the cost of lowering the number of available instances per class. We added this consideration in the study limitations.**

1. The cohen kappa score, Mathews correlation coefficients, F score values need to be evaluated.

**Reply: As evaluation metric, we chose the accuracy since the two classes were approximatively balanced (we reported in Table 1 the class distributions) and we were mainly interested in the overall correct classification (with equal weight on high and low arousal).** **We agree that additional metrics (that are mandatory for unbalanced dataset) would be very informative also for balanced datasets, providing, e.g., useful insights to which class is better classified. We added this consideration in the study limitations. **

1. Recently, the rhythm-specific deep CNN is successful for the classification of emotions using EEG signals. Could you compare with CNN-based methods?

**Reply: In the discussions we compared our results with two studies based on CNN classifiers and we identify that **

1. The advantages and disadvantages of your work must be written.

**Reply: In the discussions we added the paragraph “Study limitations”.**

# Reviewer report: Reviewer response for version 1

**DOI:** 10.5256/f1000research.24487.r92737

**Published:** 2021-09-14

**Authors:** Zahid Halim

**Affiliations:** 1 GIK Institute of Engineering Sciences and Technology, Swabi, Khyber Pakhtunkhwa, 23640, Pakistan

The paper presents an interesting approach using ML on EEG data for brain activity. Following issues need to be addressed.

1. Quantify your results in the abstract.
2. Add a gap analysis section in your introduction and also mention the research questions.
3. Fig. 1 seems generic, please add steps specific to your solution. Just a suggestion.
4. Summarize your key findings in a table in the discussion section.
5. Following references are missing
  1. On Identification of Driving-Induced Stress Using Electroencephalogram Signals: A Framework Based On Wearable Safety-Critical Scheme and Machine Learning
  2. A machine learning-based investigation utilizing the in-text features for the identification of dominant emotion in an email
  3. Imagined character recognition through EEG signals using deep convolutional neural network
  4. Investigating the use of pretrained convolutional neural network on cross-subject and cross-dataset EEG emotion recognition
  5. Cross-subject multimodal emotion recognition based on hybrid fusion
  6. A novel derivative-based classification method for hyperspectral data processing

Is the rationale for developing the new method (or application) clearly explained?

Yes

Is the description of the method technically sound?

Partly

Are the conclusions about the method and its performance adequately supported by the findings presented in the article?

Partly

If any results are presented, are all the source data underlying the results available to ensure full reproducibility?

Partly

Are sufficient details provided to allow replication of the method development and its use by others?

Yes

Reviewer Expertise:

Machine learning

I confirm that I have read this submission and believe that I have an appropriate level of expertise to confirm that it is of an acceptable scientific standard, however I have significant reservations, as outlined above.

# Response

**Published:** 2021-09-14

**Authors:** Patrizio Tressoldi

**Affiliations:** Studium Patavinum - Padova University, Italy

Thank you for your review.

 We will respond to your comments and suggestions after receiving at least a second review.

 Best wishes

 Patrizio

# Response

**Published:** 2021-10-04

**Authors:** Patrizio Tressoldi

**Affiliations:** Studium Patavinum - Padova University, Italy

1. Quantify your results in the abstract.

**Reply: We added the accuracies of the 3 best classifier-feature combinations for both the static and dynamic classifiers in the abstract *. ***

1. Add a gap analysis section in your introduction and also mention the research questions.

**Reply: We split the past introduction into two different sections: introduction and state of the art. The introduction describes the theory of the emotion, the brain anticipatory activity, the physiological correlated of the emotions and introduce the emotion recognition as a ML approach. The state of the art summarizes previous works on the emotion recognition, underling their characteristics (in terms of chosen features/classifiers and obtained performances), as well as identify the limits (i.e. performances highly variable depending from the feature/classifier combination). Finally, it describes the aims of the present work. We hope now both the limits in the literature and the aim of the present study are clearer.**

1. Fig. 1 seems generic, please add steps specific to your solution. Just a suggestion.

**Reply: This paper re-analysed previously collected data, whose details can be found in the corresponding article (ref. 37). Fig. 1 graphically summarised the experimental task of (ref. 37). We agree that, probably, it is not such informative since interest readers can look at (ref. 37) for all the details. So, we removed it.**

1. Summarize your key findings in a table in the discussion section.

**Reply: We added the table 5 to summarise the best 3 classifier/features combinations for both the static and dynamic approaches.**

1. Following references are missing
  1. On Identification of Driving-Induced Stress Using Electroencephalogram Signals: A Framework Based on Wearable Safety-Critical Scheme and Machine Learning
  2. A machine learning-based investigation utilizing the in-text features for the identification of dominant emotion in an email
  3. Imagined character recognition through EEG signals using deep convolutional neural network
  4. Investigating the use of pretrained convolutional neural network on cross-subject and cross-dataset EEG emotion recognition
  5. Cross-subject multimodal emotion recognition based on hybrid fusion
  6. A novel derivative-based classification method for hyperspectral data processing

**Reply: We added the suggested references: (1), (2), (3), (5) and (6) in the introduction, while (4) in the discussion.**
